# A Liposomal Gemcitabine, FF-10832, Improves Plasma Stability, Tumor Targeting, and Antitumor Efficacy of Gemcitabine in Pancreatic Cancer Xenograft Models

**DOI:** 10.1007/s11095-021-03045-5

**Published:** 2021-05-07

**Authors:** Takeshi Matsumoto, Takashi Komori, Yuta Yoshino, Tadaaki Ioroi, Tsukasa Kitahashi, Hiromu Kitahara, Kohei Ono, Tamami Higuchi, Masayo Sakabe, Hiroshi Kori, Masahiro Kano, Ritsuko Hori, Yukio Kato, Shinji Hagiwara

**Affiliations:** 1 Bioscience and Engineering laboratories, FUJIFILM Corporation, 577 Ushijima, Kaisei-machi, Ashigarakami-gun, Kanagawa 258-8577 Japan; 2 Analysis Technology Center, FUJIFILM Corporation, Nakanuma 210, Minamiashigara, Kanagawa 250-0193 Japan; 3 Faculty of Pharmacy, Institute of Medical, Pharmaceutical and Health Sciences, Kanazawa University, Kakuma-machi, Kanazawa, Ishikawa 920-1192 Japan

**Keywords:** liposome, gemcitabine, pancreatic cancer, dFdCTP, macrophage

## Abstract

**Purpose:**

The clinical application of gemcitabine (GEM) is limited by its pharmacokinetic properties. The aim of this study was to characterize the stability in circulating plasma, tumor targeting, and payload release of liposome-encapsulated GEM, FF-10832.

**Methods:**

Antitumor activity was assessed in xenograft mouse models of human pancreatic cancer. The pharmacokinetics of GEM and its active metabolite dFdCTP were also evaluated.

**Results:**

In mice with Capan-1 tumors, the dose-normalized areas under the curve (AUCs) after FF-10832 administration in plasma and tumor were 672 and 1047 times higher, respectively, than after using unencapsulated GEM. The tumor-to-bone marrow AUC ratio of dFdCTP was approximately eight times higher after FF-10832 administration than after GEM administration. These results indicated that liposomal encapsulation produced long-term stability in circulating plasma and tumor-selective targeting of GEM. In mice with Capan-1, SUIT-2, and BxPC-3 tumors, FF-10832 had better antitumor activity and tolerability than GEM. Internalization of FF-10832 in tumor-associated macrophages (TAMs) was revealed by flow cytometry and confocal laser scanning microscopy, and GEM was efficiently released from isolated macrophages of mice treated with FF-10832. These results suggest that TAMs are one of the potential reservoirs of GEM in tumors.

**Conclusion:**

This study found that FF-10832 had favorable pharmacokinetic properties. The liposomal formulation was more effective and tolerable than unencapsulated GEM in mouse xenograft tumor models. Hence, FF-10832 is a promising candidate for the treatment of pancreatic cancer.

**Supplementary Information:**

The online version contains supplementary material available at 10.1007/s11095-021-03045-5.

## Introduction

Pancreatic cancer is a major cause of cancer-related deaths (1). Surgical resection is a potentially curative treatment, but only a small percentage of patients undergo resection, because most diagnoses occur at an advanced stage and are therefore unresectable (2). Some chemotherapeutic agents improve the survival rate of patients with advanced pancreatic cancer. Gemcitabine (GEM)-based therapies are the first line of standard treatment; however, the clinical outcomes of this treatment have been unsatisfactory, with limited efficacy (2, 3). It is believed that the rapid clearance of GEM from systemic circulation (t_1/2_ ~ 0.3 h) is a key issue in treatment and leads to the relatively poor clinical outcomes (4, 5).

GEM, 2′,2′-difluoro 2′-deoxycytidine (dFdC), is a nucleoside analog that is effective against a number of cancer types, including pancreatic, breast, ovarian, and non-small cell lung cancers (6). GEM is taken up into cells via transporters for nucleosides, including equilibrative nucleoside transporters and concentrative nucleoside transporters (CNTs) (6), and is then metabolized to gemcitabine monophosphate. This conversion is catalyzed by deoxycytidine kinase and is the rate-determining step (7). After two more phosphates are added by other enzymes, pharmacologically active gemcitabine triphosphate (dFdCTP) is finally formed. dFdCTP blocks DNA synthesis and results in cell death (7, 8). The pharmacological effects of dFdCTP are time dependent, and sustained exposure of the tumor to the drug is required for killing cells. Cells are targeted in the synthesis phase (S phase) of the cell cycle (8–10). Because of these pharmacological and pharmacokinetic properties, a continuous infusion of GEM to produce sustained plasma levels of GEM has been investigated in clinical trials, and the findings revealed that the infusion provided longer median survival rates than those achieved using standard administration. However, using this approach, an increased incidence of hematological adverse events was observed in patients with advanced pancreatic cancer (11, 12). These findings suggested that the continuous infusion of GEM led to the accumulation of the drug in tumors over time, resulting in improved efficacy. However, the concentration of the drug was also increased in the bone marrow, resulting in increased hematological toxicity.

Liposomal encapsulation is a promising approach for the achievement of prolonged exposure of drugs. Clinically applicable carriers for liposomal formulations have been developed to protect an active pharmaceutical ingredient (API) from rapid metabolism in plasma, and to passively target the API to the tumor site by virtue of its enhanced permeability and retention (EPR) effect. The EPR effect is defined as the selective accumulation of molecules of a specific size, such as PEGylated liposomes in tumors, owing to tumor vasculature permeability (13). Although liposomal formulations of GEM have been developed to improve its stability in plasma and tumor-targeting efficiency in preclinical studies, these formulations have not yet been used clinically (4, 5, 14–17). For practical clinical use, liposomes need to be stable in plasma over the long term and be able to release a sufficient amount of GEM in the tumor tissues. However, liposome-encapsulated GEM was found to have a short half-life and to be rapidly distributed into extravascular spaces, presumably because of the instability of the formulation in plasma (5, 14, 16, 17). Thus, the construction of a stable liposomal formulation of GEM has been challenging, involving encapsulation of an extremely hydrophilic compound (5, 14–17).

Our liposomal formulation of GEM, named FF-10832, was designed to have long-term stability in plasma, high accumulation in tumors, and payload release at a rate that maintains GEM concentrations in the therapeutic range for optimal periods of time. The aim of the study was to characterize the pharmacokinetic properties and compare the in vivo antitumor efficacies of a liposomal GEM formulation with those of unencapsulated GEM. In this study, FF-10832 was observed to have strong antitumor effects without severe body weight loss in mice with subcutaneous xenografted Capan-1 tumors, which are GEM-sensitive (18), and BxPC-3 tumors, which are GEM-insensitive (19), as well as in mice with orthotopic xenografted SUIT-2 tumors. We attempted to determine the mechanism underlying the enhanced antitumor activity. Our findings provide evidence supporting the validity of an ongoing clinical Phase 1 study for the treatment of pancreatic cancer using this novel liposomal formulation.

## Materials and Methods

### Materials

Hydrogenated soy phosphatidylcholine (HSPC) and N-(methylpolyoxyethylene oxycarbonyl 2000)-1,2-distearoyl-*sn*-glycero-3-phosphoethanolamine, sodium salt (N-MPEG-2000-DSPE) were procured from NOF Corporation (Tokyo, Japan). Gemcitabine hydrochloride was procured from Plantex Ltd. (Netanya, Israel). Cholesterol was procured from Nippon Fine Chemical (Osaka, Japan). Ethanol, ethyl acetate, sodium chloride, disodium hydrogen phosphate 12 hydrate, sodium dihydrogen phosphate dihydrate, sucrose, and l-histidine were procured from Merck KGaA (Darmstadt, Germany). Sodium hydroxide solution (8 mol/L) was purchased from Wako Pure Chemicals (Osaka, Japan). 1,1′-dioctadecyl-3,3,3′,3′-tetramethylindocarbocyanine perchlorate (DiI) and 1,1′- dioctadecyl-3,3,3′,3′-tetramethylindotricarbocyanine iodide (DiR) were purchased from Thermo Fisher Scientific (Waltham, MA). Gemcitabine triphosphate and 3,3′-diaminobenzidine were purchased from Sigma-Aldrich (St. Louis, MO).

### Preparation of Lipid Encapsulated FF-10832 and Labeled FF-10832

FF-10832 was prepared as previously reported (20). Briefly, an empty liposome containing cholesterol, HSPC, and N-MPEG-DSPE at a molar ratio of 4:15:1 was first prepared. GEM was loaded into the liposome by the passive-loading method, and then unencapsulated GEM was removed by diafiltration. The purified liposome was sterilized by filtration. In a previous report, no transfer of fluorescence from liposomes, which consists of HSPC/cholesterol/N-MPEG-DSPE/DiR, to lipoproteins or other plasma proteins appeared, and the dye was believed to be stably incorporated within the liposomes in vivo when the fluorescence dye (DiR) was added while preparing the liposomes (21). Therefore, labeled FF-10832 (FF-10832-DiI and FF-10832-DiR) was prepared by adding DiI or DiR while preparing the liposomes used in the present study. FF-10832-DiI and FF-10832-DiR consist of cholesterol/HSPC/N-MPEG-DSPE/DiI or DiR at a molar ratio of 4:15:1:0.04. Details are provided in the Supplementary Methods.

### Morphology, Size, and Stability Assays

The morphology and size of FF-10832 were assessed using transmission electron microscopy (cryo-TEM, JEM-2010, JEOL Ltd., Tokyo, Japan) and dynamic light scattering (DLS, ELSZ-2000ZS, Otsuka Electronics, Tokyo, Japan), respectively. Stability was checked according to the guidelines recommended by the International Federation of Pharmaceutical Manufacturers & Associations (22), under storage conditions of 5°C ± 3°C for up to 24 months. The encapsulation efficiency (%) was calculated as follows:

1 $$ \mathrm{Encapsulation}\ \mathrm{efficiency}\ \left(\%\right)=\left({\mathrm{C}}_{\mathrm{total}}-{\mathrm{C}}_{\mathrm{free}}\right)/{\mathrm{C}}_{\mathrm{total}}\times 100 $$

where C_total_ and C_free_ represent the concentrations of total and unencapsulated GEM, respectively. To prepare samples for measuring unencapsulated GEM, 200 μL of FF-10832 containing approximately 0.5 mg/mL of GEM were loaded onto an ultrafilter (Amicon® Ultra-0.5, MWCO: 10 K, Merck Millipore, MA) and centrifuged (7400×g, 20°C, 30 min). The concentrations of GEM and total lipids were analyzed using high-performance liquid chromatography (HPLC) and HPLC/charged aerosol detector (HPLC-CAD), respectively. The details of the procedures are provided in the Supplementary Methods. Experiments at each time point were performed in triplicate.

### Animals

All animal studies were conducted in compliance with the “Act on Welfare and Management of Animals” and Code of Ethics for Laboratory Animals of Fujifilm Corporation. For subcutaneous mouse xenograft models, Capan-1 and BxPC-3 pancreatic cancer cells were passaged in a non-confluent state, and 3–10 × 10^6^ Capan-1 cells and 1 × 10^7^ BxPC-3 cells were suspended in 100 μL of fetal bovine serum (FBS)-free medium (Capan-1: IMDM, BxPC-3: RPMI 1640) and subcutaneously injected into the right flank of female BALB/cAJcl-nu/nu mice (CLEA Japan, Kanagawa, Japan). SUIT-2 pancreatic cancer cells were orthotopically administered as previously described (23). Briefly, the cells (1 × 10^7^) suspended in 10 μL of FBS-free RPMI 1640 (Fujifilm Wako Pure Chemical Corp., Osaka, Japan) medium were inoculated into the pancreas of female BALB/cAJcl-nu/nu mice that were anesthetized with isoflurane. For control groups, corresponding FBS-free medium was similarly injected.

### In Vivo Pharmacokinetics

FF-10832-DiI was confirmed to be stable in plasma for 72 h in preliminary in vitro studies (data not shown). Therefore, DiI was chosen to label FF-10832 for in vivo pharmacokinetic analysis. Female BALB/cAJcl nu/nu mice were intravenously administered FF-10832 (4 mg/kg) and GEM (240 mg/kg) via the tail vein. Mice with Capan-1 or BxPC-3 tumors (*n* = 4 animals/group) were intravenously administered FF-10832 (4 mg/kg), FF-10832-DiI (4 mg/kg), GEM (240 mg/kg), or a vehicle (9.4% sucrose solution) via the tail vein, when the average tumor volume reached approximately 100–500 mm^3^. Both FF-10832 and labeled FF-10832 at a dose of 4 mg/kg of GEM contained 125 mg/kg of phospholipids. To measure plasma concentrations of GEM in female BALB/cAJcl nu/nu mice, 600 μL of blood was collected 0.25, 2, 4, 8, 24, and 48 h after FF-10832 administration, 50 μL of blood was collected 5 min and 0.25, 0.5, 2, 4, and 24 h after GEM administration. The blood was added to tubes containing tetrahydrouridine (THU), an inhibitor of cytidine deaminase, at a final concentration of 100 μg/mL and centrifuged for 800×g for 10 min at 4°C to prepare the plasma samples. The plasma samples were stored at −40°C until analysis. To measure the tissue concentrations of GEM and dFdCTP, tumor and bone marrow tissues were collected 4, 24, 32, 48, and 72 h after administration of GEM or FF-10832. For in vivo GEM release, tumor tissues and blood samples were collected 6, 24, and 48 h after FF-10832-DiI administration. Tumor and bone marrow tissues were harvested, placed in tubes containing THU at a final concentration of 100 μg/g tissue, weighed, flash frozen in liquid nitrogen, and stored at −80°C until analysis.

The concentrations of GEM and dFdCTP were analyzed using liquid chromatography/tandem mass spectrometry, and concentrations of DiI were analyzed using HPLC. Details of the procedures are provided in the Supplementary Methods section.

GEM release (%) was indirectly calculated using a previously published formula (24), with modifications:

2 $$ \mathrm{GEM}\ \mathrm{release}\ \left(\%\right)=\left\{1-\left[\mathrm{Gem}\ \left(\mathrm{t}\right)/\mathrm{DiI}\ \left(\mathrm{t}\right)\right]/\left[\mathrm{Gem}\ (0)/\mathrm{DiI}\ (0)\right]\right\}\times 100 $$

where [Gem (0)] and [DiI (0)] represent the GEM and DiI concentrations, respectively, in the solution administered to the animals, and [Gem (t)] and [DiI (t)] represent GEM and DiI concentrations, respectively, in plasma and tumor tissues at time *t* after administration. In developing this equation, we assumed that total GEM concentrations were comparable to encapsulated GEM concentrations in plasma and tumors, because unencapsulated GEM could quickly disappear due to its metabolic instability and rapid tissue distribution. In fact, the concentration of unencapsulated GEM measured using ultrafiltration in plasma after the administration of FF-10832 at 4 mg/kg (Fig. S1) were less than 1/100 of the corresponding total GEM concentrations.

### Whole-Body Imaging

The near infrared dye DiR, which has low tissue autofluorescence (21), was chosen for whole-body imaging. Mice with Capan-1 tumors were intravenously administered FF-10832-DiR (3 mg/kg) via the tail vein. Whole-body imaging was performed 72 h after administration using an ImageQuant LAS 4000 image reader (GE Healthcare, Chicago, IL) after mice were anesthetized with a 2.5% isoflurane/air mixture in a plastic chamber.

### Histology and Flow Cytometric Analysis

Mice with Capan-1 or BxPC-3 tumors were administered FF-10832-DiI (4 mg/kg) or vehicle via the tail vein. For histology, the tumors were collected 72 h after administration and snap-frozen for cryo-sectioning. The frozen tissue sections were imaged using a fluorescence microscope (OLYMPUS, Tokyo, Japan) to detect FF-10832-DiI. Vascular endothelial cells in the sections were subsequently stained with rat anti-mouse CD31 antibody (×100 diluted BD Pharmingen™ #550274, BD Biosciences, Franklin Lakes, NJ), followed by staining with 3,3′-diaminobenzidine and hematoxylin–eosin.

For flow cytometric analyses, single-cell suspensions of the tumors were prepared using gentle MACS (Miltenyi Biotec, North Rhine-Westphalia, Germany), and the cells were stained with BV421-conjugated anti-mouse F4/80 antibody (BD Biosciences), and APC-conjugated anti-human epithelial cell adhesion molecule (EpCAM/CD326) antibody (BD Biosciences). Flow cytometric analyses were performed using a FACSAriaIII (BD Biosciences).

### Confocal Laser Scanning Microscopy Analysis

Mice with Capan-1 tumors were administered FF-10832-DiI (4 mg/kg) or the vehicle via the tail vein. The tumors were collected 24 h after administration. Single-cell suspensions of the tumors were prepared using gentle MACS. The single-cell suspensions were purified using F4/80 MicroBeads (Miltenyi Biotec). Briefly, the F4/80-positive cells were magnetically labeled with anti-F4/80 MicroBeads. The cell suspension was then loaded onto a MACS LS Column (Miltenyi Biotec). After removing the column from the magnetic field, the magnetically retained F4/80-positive cells were eluted. The purified cells (2 × 10^5^ cells/well) were seeded into 96-well plates (CellCarrier-96, PerkinElmer, Waltham, MA), and stained for 30 min with 50 nM LysoTracker Deep Red (Thermo Fisher Scientific) in RPMI 1640 containing 10% FBS and 1% Penicillin–Streptomycin. The cells were then stained for 10 min with 10 μg/mL Hoechst 33342 (Dojindo Laboratories, Kumamoto, Japan) in phosphate buffered saline (PBS). The cell images were captured using a confocal quantitative image cytometer CQ1 (Yokogawa Electric, Tokyo, Japan) and analyzed using the CQ1 proprietary measurement software. All experiments were performed in triplicate.

### In Vitro GEM Release from Peritoneal Macrophages Internalizing FF-10832

Male Jcl:ICR mice (CLEA Japan, Kanagawa, Japan) were intraperitoneally administered 5 mg/kg of FF-10832. Three hours after administration, 10 mL of ice-cold PBS was administered into the peritoneal cavity, and peritoneal fluids were collected for isolation. After washing twice with PBS followed by centrifugation at 400×g for 5 min at 4°C, the cells were suspended in RPMI 1640 medium, seeded into a 96-well plate, and incubated at 37°C in a 5% CO_2_ atmosphere. Subsequently, the cells were centrifuged at 500×g for 5 min at 4°C, the medium was ultrafiltered at 14,000×g for 15 min at 4°C using an Amicon® Ultra-0.5, and the supernatants were collected for the determination of GEM concentration using LC-MS/MS at 0, 0.25, 0.5, 1, 3, 6, and 24 h after seeding. The GEM release (%) was calculated as the ratio of drug concentration in the supernatant to that in the cells before seeding × 100.

### In Vivo Antitumor Activity

Female BALB/cAJcl-nu/nu mice with Capan-1 or BxPC-3 tumors were randomized and intravenously administered either FF-10832 (2, 3, 4, or 5 mg/kg), GEM (240 mg/kg), or the vehicle (9.4% sucrose solution) once a week for 3 weeks. The length and width of the tumors were measured twice a week. The tumor volume was obtained as follows:

3 $$ \mathrm{Tumor}\ \mathrm{volume}=\mathrm{length}\times {\left[\mathrm{width}\right]}^2\times 0.5 $$

SUIT-2 cells, a human pancreatic cancer cell line, was used as an orthotopic tumor model. Seven days after the implantation of SUIT-2 cells, mice were randomized and 240 mg/kg GEM, 4 mg/kg FF-10832, or 9.4% sucrose solution was intravenously administered via the tail vein once a week for 11 weeks. The mice were followed for 91 days after implantation, and the event-free survival (EFS) time from tumor transplantation until death or moribundity were evaluated. Moribundity included >20% decrease in body weight, hypothermia, or other conditions requiring euthanasia.

### Detection of Inhibition of DNA Synthesis

Female BALB/cAJcl-nu/nu mice with Capan-1 or BxPC3 tumors were intravenously administered FF-10832 (4 mg/kg), GEM (240 mg/kg), or vehicle (9.4% sucrose solution) via the tail vein. The tumors were collected, sectioned, and stained using Click-iT EdU Alexa Fluor 488 Imaging Kits (Thermo Fisher Scientific). Images were captured on a confocal quantitative image cytometer CQ1 (Yokogawa Electric), and the percentage of EdU-positive DNA-synthesizing cells was calculated and normalized by the value measured after vehicle administration. Details of the procedures are provided in the Supplementary Methods section.

### Statistical Analysis

Median fluorescence intensity was assessed 72 h after administration using Tukey’s multiple comparison test for the median of all groups. Tumor volumes were assessed on each evaluation day using Tukey’s multiple comparison test for the means of all groups. The EFS was graphically represented using Kaplan–Meier analysis, and the EFS between groups were compared using log-rank tests in GraphPad Prism (GraphPad Software, San Diego, CA). Differences with *p* < 0.05. were considered to be statistically significant.

## Results

### Morphology, Mean Particle Size, and Stability of FF-10832

**Fig. 1:**
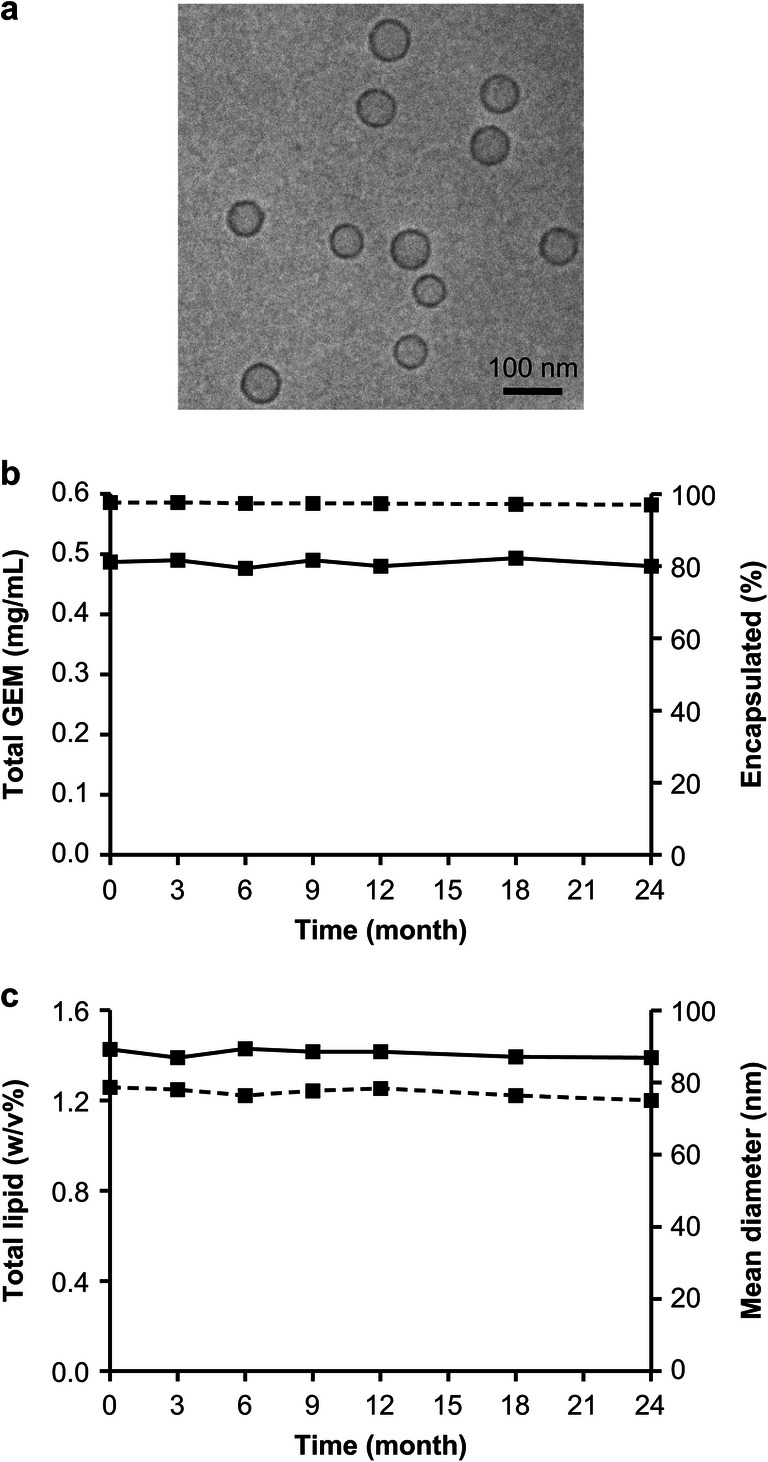
**Characterization of FF-10832.** (**a**) TEM images of FF-10832. The scale bars represent 100 nm. (**b**) Total GEM (bold line) and encapsulation rate (%) of GEM (dashed line) of FF-10832 during storage for 24 months at 5°C ± 3°C are shown. (**c**) The total lipid (bold line) and mean particle diameter (dashed line) of FF-10832 during storage for 24 months at 5°C ± 3°C are shown. Each data point represents the mean ± standard deviation (*n* = 3)

TEM analysis revealed that FF-10832 was homogeneous in appearance, and consisted of unilamellar vesicles (Fig. 1a). The total GEM content, total lipid content, and particle size were 0.49 ± 0.01 (mg/mL), 1.43 ± 0.01 (*w*/*v*%), and 79 ± 2 (nm), respectively, and these results were stable during the 24-month storage at 5°C ± 3°C (Figs. 1b, c). This stability allowed >96% encapsulation of the drug (Fig. 1b). These stability data suggested that FF-10832 is suitable for clinical applications.

### Pharmacokinetics of FF-10832 in Mice with Capan-1 Tumors

Although we have reported plasma and tumor concentrations of GEM in a mouse model of Colon26 peritoneal dissemination after FF-10832 administration (20), the detailed pharmacokinetics of FF-10832 in mice with human pancreatic tumors are still unclear. The plasma and tumor concentration profiles of GEM were measured in mice with Capan-1 tumors after administration of FF-10832 at a dose of 4 mg/kg, and compared with those in mice after administration of GEM at a dose of 240 mg/kg. The doses selected for FF-10832 and GEM corresponded to the maximum tolerated doses (MTDs), which induced body weight loss of 20%.

**Fig. 2:**
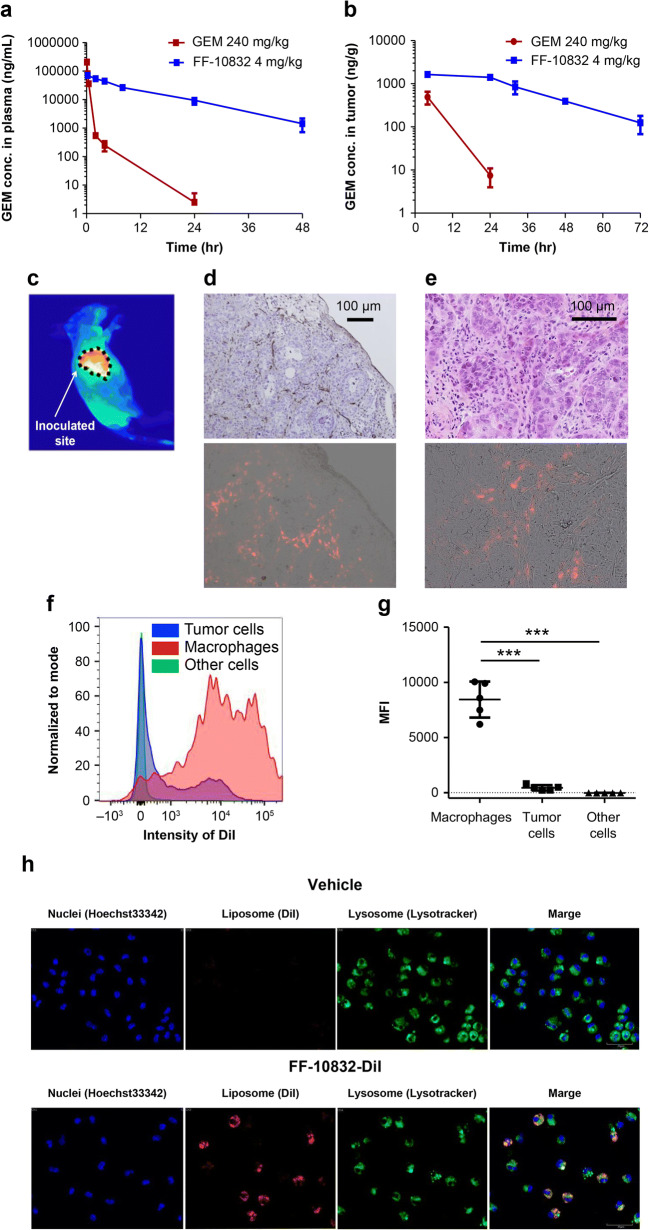
**Pharmacokinetics of FF-10832.** (a and b) Plasma (**a**) and tumor (**b**) concentrations of GEM in mice with Capan-1 tumors after a single intravenous dose of FF-10832 (blue, 4 mg/kg) or GEM (brown, 240 mg/kg) are shown. Each data point represents the mean ± standard deviation (*n* = 4 animals/time point). (**c**–**e**) Distribution of FF-10832 labeled with a fluorescent dye (FF-10832-DiR or FF-10832-DiI) at 72 h after a single intravenous dose in mice with Capan-1 tumors is shown. Whole-body imaging of FF-10832-DiR (**c**) DiI-fluorescence (red) imaging (lower d and e) of a tumor cryosection stained with CD31 detecting blood vessels (upper d) or hematoxylin and eosin staining (upper e) are shown. The scale bars represent 100 μm. (**f**, **g**) Distribution of FF-10832-DiI in EpCAM- and F4/80-positive cells in Capan-1 tumors 72 h after a single intravenous dose. Flow cytometric histogram plots (**f**) for EpCAM-positive (tumor, blue), F4/80-positive (macrophages, red), and other (green) cells, and fluorescence intensity histogram of DiI in each fraction are shown. The y axis shows the relative cell count for each population (normalized to mode), and the x axis shows the DiI-fluorescence intensity. Comparison of the distribution of FF-10832-DiI between F4/80-positive cells (macrophages), EpCAM-positive (tumor) cells, and other cells (**g**). Each data point represents the median ± standard deviation (*n* = 5 animals/group). *** *p* < 0.001 vs. tumor cells, and *** *p* < 0.001 vs. other cells. (**h**) Uptake of FF-10832-DiI in F4/80-positive cells in Capan-1 tumors is shown by confocal laser scanning microscopy images for nuclei (blue), DiI (red), lysosome (green), and merged

In plasma, GEM concentrations >1000 ng/mL were observed for up to 0.5 h after GEM administration, and up to 48 h after FF-10832 administration (Fig. 2a). The pharmacokinetic parameters (V_0_, AUC_0-last_, CL, V_0_, Vd_ss_, and t_½_) of GEM after administration of FF-10832 or GEM are shown in Table I. Compared with the administration of unencapsulated GEM, the administration of FF-10832 decreased CL 675-fold, V_0_ 13-fold, and Vd_ss_ 26-fold. The parameter t_½_ was increased three-fold and AUC_0-last_/dose was increased 672-fold.

**Table I:** Pharmacokinetic parameters of GEM after FF-10832 and GEM intravenous administration

	Parameters	Unit	GEM	FF-10832
			240 mg/kg^a)^	4 mg/kg^a)^
Plasma	AUC_0-last_	μg·h/mL	73.0	821
	CL	mL/min/kg	54.8	0.0812
	V_0_	L/kg	0.722	0.0545
	V_dss_	L/kg	1.40	0.0592
	t_1/2_	h	2.90	8.18
	AUC_0-last_ / Dose	min·kg/mL	0.0183	12.3
Tumor	AUC_0-last_ / Dose	min·kg/g	0.000824	0.863

All the parameters were calculated for mean values of GEM concentration profile (n = 4 animals/each time point)

a) Dose equivalent to GEM

Similarly, longer exposure of Capan-1 tumors to >100 ng/g GEM was achieved for up to 72 h after FF-10832 administration, but only for <24 h after GEM administration (Fig. 2b). The AUC_0-last_/dose in tumors after FF-10832 administration was increased 1047-fold relative to that after GEM administration (Table I).

These results indicated that systemic and tumor exposures were higher for GEM FF-10832 than for GEM itself in mice with tumors.

### Uptake of FF-10832 in Capan-1 Tumors

To understand the localization of FF-10832 in mice with tumors, GEM-encapsulated liposomes incorporating DiI and DiR into the lipid bilayer (FF-10832-DiI and FF-10832-DiR, respectively) were intravenously administered to mice with tumors, and fluorescence was monitored. HPLC analysis revealed a gradual increase in tumor DiI concentration until 24 h, which was then constant for up to 72 h after FF-10832-DiI administration (data not shown). On the basis of these results, whole-body imaging was performed 72 h after FF-10832-DiR administration, and found that FF-10832-DiR was distributed in tumors (Fig. 2c). Then, to confirm the localization of the liposome within the tumor microenvironment, immunohistochemical staining using CD31 antibodies of tumor blood vessels was performed with concomitant hematoxylin and eosin staining. When stained images were compared with the DiI images in sequential cryosections, FF-10832 was shown to primarily accumulate in the tumor stroma outside of the tumor vessels, and minimal accumulation was observed in the tumor cells (Figs. 2d, e). The localization of FF-10832 in tumors was further analyzed using fluorescence-activated cell sorting, in which F4/80 and EpCAM were used as marker antigens for macrophages and tumor cells, respectively. F4/80-positive cells isolated from Capan-1 tumors exhibited strong DiI signals, whereas EpCAM-positive cells or other cells did not (Fig. 2f). The DiI signals were significantly (*p* < 0.001) more intense in F4/80-positive cells than in EpCAM-positive cells or other cells (Fig. 2g). Confocal fluorescence microscopy analysis revealed that FF-10832-DiI (red florescence) co-localized with lysosomal markers (green fluorescence) in F4/80-positive cells (Fig. 2h). Overall, these results suggested that FF-10832 was extravasated from the blood into tumors, possibly by the EPR effect, where they were at least partially internalized, and then processed in lysosomes in TAMs.

### Payload Release of FF-10832

**Fig. 3:**
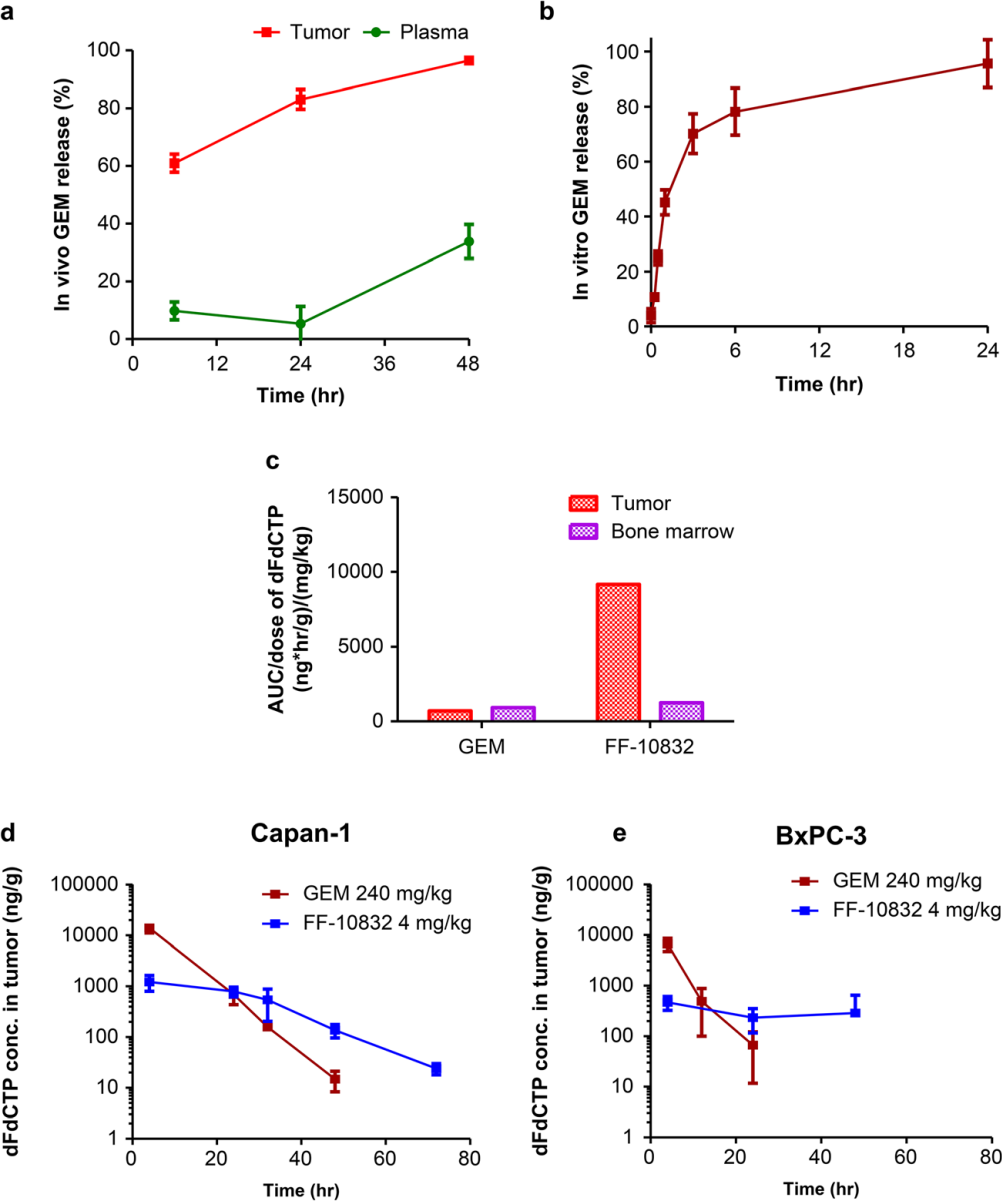
**Release of GEM and Appearance of dFdCTP After Administration of FF-10832.** (**a**) In vivo GEM releases (%) in plasma (green) and tumor tissue (red) of mice with Capan-1 tumors after a single intravenous dose of FF-10832-DiI. Concentrations of GEM and DiI were measured in plasma, tumor, and the administered solution, and the percentage release of GEM was calculated according to Eq. (2). Each data point represents the mean ± standard deviation (*n* = 6 animals/time point). (**b**) In vitro GEM release (%) from peritoneal macrophages isolated from mice treated with a single intraperitoneal dose of FF-10832. The percentage of GEM released was calculated as described in the Materials and Methods section. Each data point represents the mean ± standard deviation (*n* = 4 animals/time point). (**c**) The mean dose-normalized AUC_0-last_ results of dFdCTP in Capan-1 tumors (red) and bone marrow (purple) of mice are shown for mice with Capan-1 tumors treated with a single intravenous dose of 240 mg/kg GEM and 4 mg/kg FF-10832. The dFdCTP concentrations were calculated at 4, 24, 32, 48, and 72 h after administration, and the AUC_0-last_/dose values were calculated. The parameters were calculated for mean values of the dFdCTP concentration profile (*n* = 4 animals/each time point). (**d**, **e**) Concentrations of dFdCTP in Capan-1 (**d**) and BxPC-3 (**e**) tumors after a single intravenous dose of GEM (brown) and FF-10832 (blue). Each data point represents the mean ± standard deviation (*n* = 4 animals/time point)

We attempted to measure the payload released from liposomes in tumor tissues. However, it was technically difficult to directly measure the GEM released from liposomes in vivo, so the ratios of released GEM in plasma and tumors were calculated based on the concentrations of DiI and GEM in plasma, tumor, and the administered solution, according to Eq. (2). The release of GEM after intravenous administration of FF-10832-DiI at a dose of 4 mg/kg was higher in tumors than in plasma (Fig. 3a).

To examine whether macrophages in which FF-10832 was loaded and internalized could release GEM to the extracellular space, FF-10832 was intraperitoneally administered to mice at a dose of 5 mg/kg, and peritoneal macrophages were isolated 3 h after administration. A confocal laser scanning microscopy analysis revealed that FF-10832-DiI (red florescence) was at least partially co-localized with lysosomal markers (green fluorescence) in peritoneal macrophages (Fig. S2). The concentrations of GEM were then examined in the culture supernatant over time. GEM was time-dependently released from the isolated macrophages, and the amount of GEM in the supernatant reached 96% of the internalized GEM in macrophages after 24 h in vitro (Fig. 3b). These results supported our hypothesis that GEM can be released from macrophages into the extracellular space, such as in a tumor microenvironment, when FF-10832 is administered into mice, although quantitative estimation of the contribution of such release from macrophages to overall GEM exposure to tumor cells is still difficult. The released GEM might be taken up by the surrounding cells via membrane transporters.

### Concentration of an Active Metabolite GEM (dFdCTP) after Administration of FF-10832 in Mice with Capan-1 Tumors and BxPC-3 Tumors

After systemic administration of GEM, the drug was distributed to tissues non-selectively and relatively uniformly (25), leading to adverse myelosuppression, in addition to favorable antitumor activity. Liposome-encapsulated GEM was expected to increase tumor exposures via the EPR effect, reducing the exposures of normal tissue to GEM. Higher levels of GEM in the tumors were observed after FF-10832 administration than after GEM administration (Fig. 2b). To confirm the selective distribution of the active API, GEM, in the tumor microenvironment, dFdCTP concentrations in both Capan-1 tumors and bone marrow were measured (Fig. 3c). After the administration of FF-10832, the tumor-to-bone marrow AUC ratio was higher for dFdCTP (AUC ratio, 8.5) than that for GEM (AUC ratio, 1.1). It therefore appeared feasible that FF-10832 could have enhanced antitumor effects, with reduced myelosuppression.

Capan-1 tumors were experimentally sensitive (18), whereas BxPC-3 tumors were insensitive to GEM (19) in vivo. BxPC-3 tumors have been reported to express equilibrative nucleoside transporter 1 (ENT1), which is involved in the cellular uptake of GEM, at low levels (26). The concentrations of dFdCTP in tumors were examined in mice with Capan-1 or BxPC-3 tumors after administration of 4 mg/kg of FF-10832 or 240 mg/kg of unencapsulated GEM (Figs. 3d, e). In both tumor models, GEM administration resulted in higher initial dFdCTP concentrations followed by rapid reduction, whereas FF-10832 administration produced sustained dFdCTP concentrations (Figs. 3d, e). The pharmacokinetic parameters of dFdCTP in BxPC-3 and Capan-1 tumors are shown in Table II. After GEM administration, C_max_, AUC_0-last_, and MRT_0-last_ of dFdCTP in BxPC-3 tumors were decreased 2.1-fold, 3.4-fold, and 1.4-fold, respectively, relative to those in Capan-1 tumors (Table II). This observation was compatible with the lower expression of GEM uptake transporter in BxPC-3 tumors (26). After FF-10832 administration, the C_max_ and AUC_0-last_ in BxPC-3 tumors were decreased 2.6-fold and 2.4-fold, respectively, relative to those in Capan-1 tumors, whereas the MRT_0-last_ values were comparable between BxPC-3 and Capan-1 tumors (Table II). These results indicated that BxPC-3 had a lower exposure to dFdCTP than Capan-1 tumors after administration of FF-10832 and GEM, although the duration of exposure after FF-10832 administration was comparable between BxPC-3 and Capan-1 tumors (Figs. 3d, e). The concentrations of dFdCTP for 48 h after FF-10832 administration were also comparable between BxPC-3 and Capan-1 tumors (Figs. 3d, e).

**Table II:** Pharmacokinetic parameters of an active metabolite of GEM dFdCTP in Capan-1 and BxPC-3 tumors

Parameters	Unit	GEM 240 mg/kg^d)^		FF-10832 4 mg/kg^d)^	
		Capan-1	BxPC-3	Capan-1	BxPC-3
C_max_^a)^	μg/g	13.9	6.66	1.21	0.467
AUC_0-last_^b)^	μg·h/g	120	35.1	33.7	13.9
MRT_0-last_^c)^	h	8.95	6.31		23.2

All the parameters were calculated for mean values of dFdCTP concentration profile (n = 4 animals/each time point)

a) Maximum dFdCTP concetration in tumor

b) AUC up to last measured concentration time point in tumor

c) Mean residence time up to last measured concentration time point in tumor

d) Dose equivalent to GEM

### Antitumor Activity of FF-10832 in Mouse Subcutaneous Xenograft Tumor Models

**Fig. 4:**
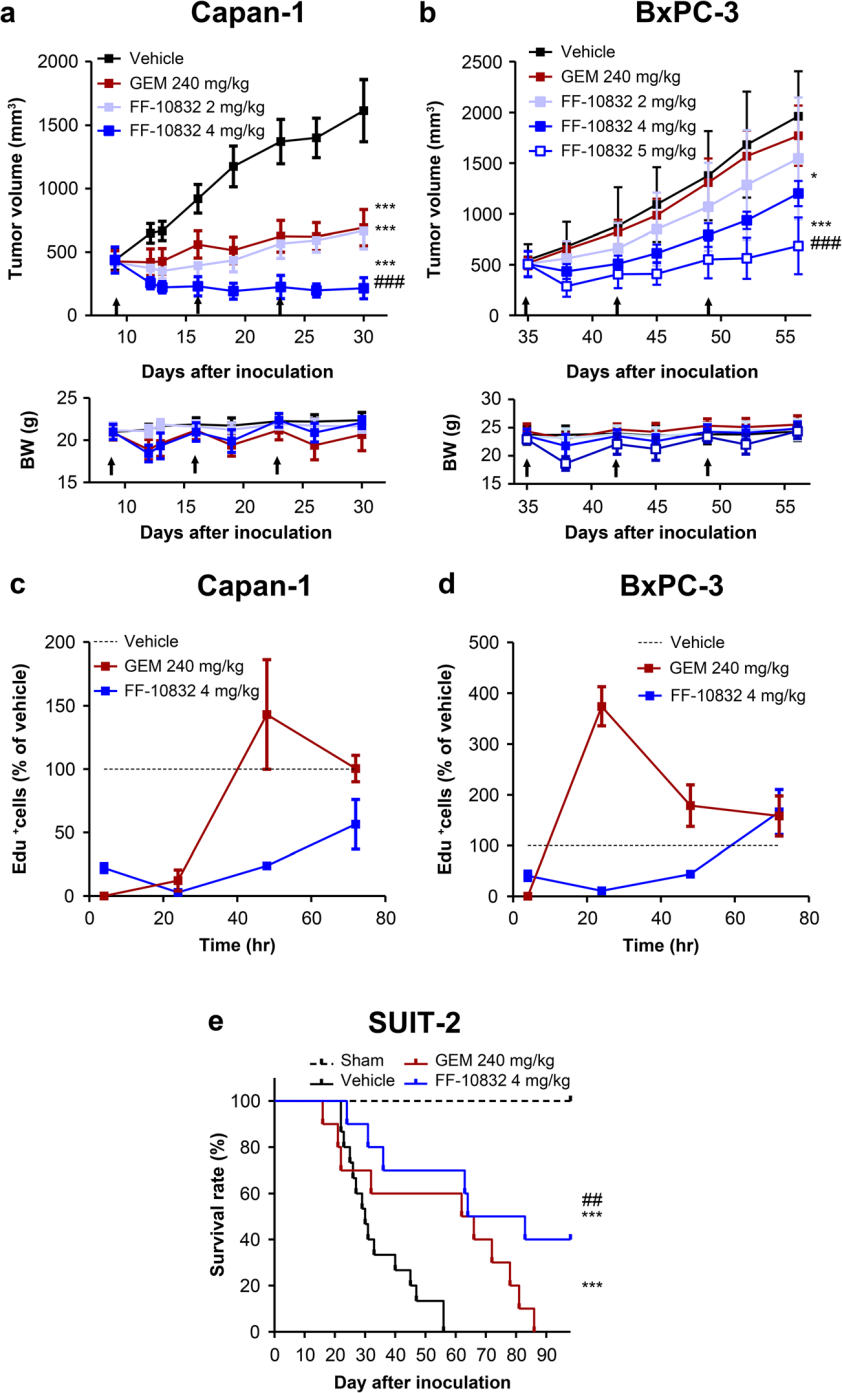
**Pharmacological Efficacy of FF-10832.** Mice with Capan-1 (**a**) or BxPC-3 (**b**) tumors were intravenously treated with the vehicle (black), 240 mg/kg of GEM (brown), or 2 mg/kg (light blue), 4 mg/kg (blue), and 5 mg/kg (blue white) of FF-10832 once a week for 3 weeks (indicated as arrows), and tumor volumes that were measured over time are shown in the upper graphs, with changes in body weights in the lower graphs. Each data point represents the mean ± standard deviation (*n* = 8 animals/group). * *p* < 0.05, *** *p* < 0.001 vs. vehicle, and ### *p* < 0.001 vs. GEM. Inhibition of DNA synthesis in Capan-1 (**c**) and BxPC-3 tumors (**d**) after a single intravenous dose of FF-10832 (blue) and GEM (brown). DNA synthesis was evaluated by using the EdU incorporation assay, and the fraction of EdU-positive cells were measured and normalized to that after vehicle administration. Each data point represents the mean ± standard deviation (*n* = 3 animals/time point). The survival rates of mice with SUIT-2 tumors intravenously treated with vehicle (black), 240 mg/kg of GEM (brown), or 4 mg/kg of FF-10832 (blue) once a week for 11 weeks are shown (**e**), with significant differences in EFS times; *** *p* < 0.001 vs. vehicle, and ## *p* < 0.01 vs. GEM (*n* = 8/group)

To evaluate their antitumor activities in mouse subcutaneous xenograft models, FF-10832 or GEM at doses up to the MTD were intravenously administered to mice with Capan-1 or BxPC-3 tumors once a week for 3 weeks. In mice with Capan-1 tumors, tumor growth was significantly (*p* < 0.001) suppressed by FF-10832 and GEM relative to the vehicle control (Fig. 4a). FF-10832 at 2 mg/kg and 4 mg/kg exhibited dose-dependent antitumor activity, and at 4 mg/kg was significantly higher (*p* < 0.001) than that of GEM at the MTD (240 mg/kg; Fig. 4a). Similar body weight decreases were transiently observed after each drug treatment in mice treated with FF-10832 at 4 mg/kg and GEM at 240 mg/kg.

In mice with BxPC-3 tumors, unencapsulated GEM at MTD did not suppress tumor growth (Fig. 4b). In contrast, significant suppression of tumor growth was observed for FF-10832 at 4 mg/kg and 5 mg/kg (*p* < 0.05 and *p* < 0.001, respectively) relative to that of the vehicle (Fig. 4b). A transient body weight decrease, which was evident in comparison with the vehicle control, was observed in the FF-10832 5 mg/kg group and was limited to the first dosing.

These results indicated that the antitumor efficacy of FF-10832 was superior to that of unencapsulated GEM in these subcutaneous xenograft tumor models.

### Pharmacodynamic Analysis of FF-10832 in Mice with Capan-1 and BxPC-3 Tumors

To support prolonged target-organ exposure to FF-10832, mice with Capan-1 or BxPC-3 tumors received FF-10832 intravenously at 4 mg/kg or GEM at 240 mg/kg, and the time profiles of EdU labeling of the tumor tissues were examined (Figs. 4c, d). EdU labeling was inhibited for 72 and 48 h after FF-10832 administration to Capan-1 and BxPC-3 tumors, respectively, whereas EdU labeling was initially inhibited, but reverted to be higher than the vehicle control level and peaked at 48 and 24 h after GEM administration in Capan-1 and BxPC-3 tumors, respectively (Figs. 4c, d). This inhibition of EdU labeling over time appeared to be compatible with more sustained dFdCTP concentrations after the administration of FF-10832 than that with the administration of unencapsulated GEM in each tumor (Figs. 3d, e).

### Antitumor Activity of FF-10832 in a Mouse Orthotopic Xenograft Tumor Model

To evaluate antitumor activities in mouseorthotropic xenograft pancreatic tumors, SUIT-2 cells were selected. SUIT-2 orthotropic transplantation reportedly causes pancreatic adenocarcinoma that mimics the pattern of disease progression of human pancreatic cancer (23). FF-10832 at 4 mg/kg or GEM at 240 mg/kg was intravenously administered once a week for 11 weeks, and the survival times were compared (Fig. 4e). The median survival times in the FF-10832 and GEM groups were 89 and 74 days, respectively, and were significantly longer than those in the vehicle control group (36.5 days), with increased EFS times (both *p* < 0.001). The EFS time in the FF-10832 group was significantly increased relative to that in the GEM group (*p* < 0.01). The survival rates until termination of the experiment were 0% for the vehicle and GEM groups and 40% for the FF-10832 group. These results indicated that the antitumor efficacy of FF-10832 was superior to that of GEM in a clinically relevant mouse model.

## Discussion

**Fig. 5:**
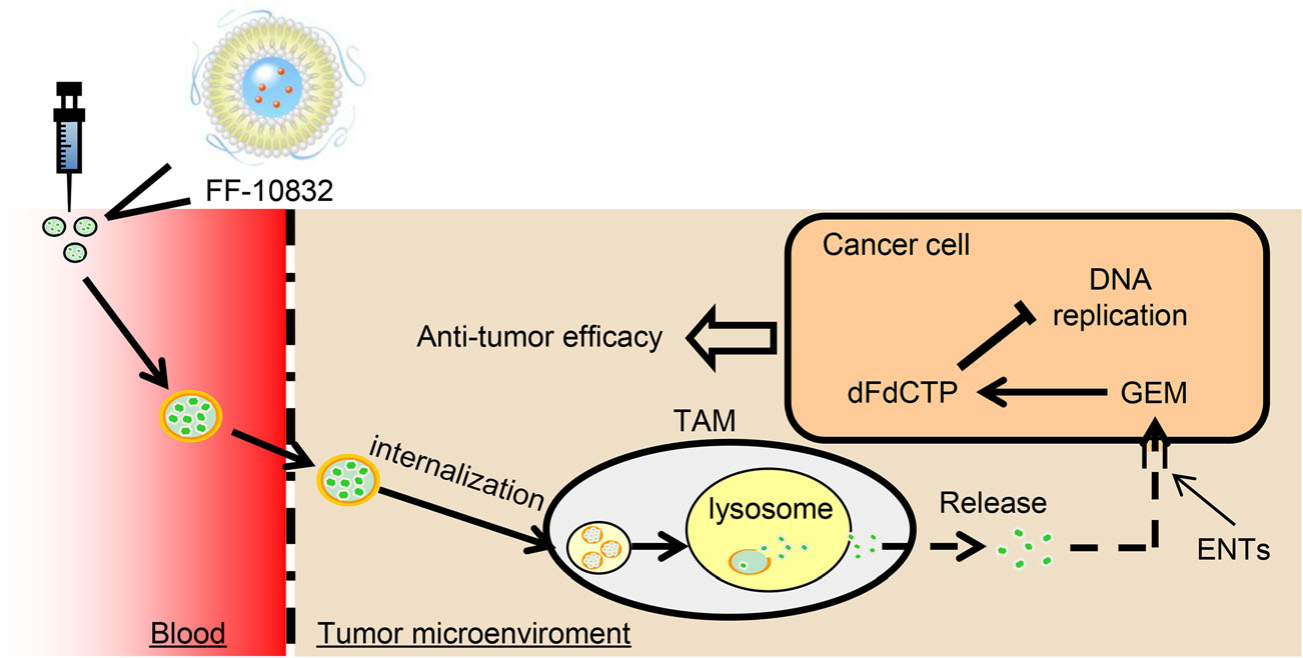
**Summary of FF-10832.** The postulated antitumor efficacy of FF-10832 is illustrated. FF-10832 is extravasated from blood into tumors, possibly by the EPR, and internalized in TAMs, in which FF-10832 is processed in lysosomes to release GEM. The released drug is imported into cancer cells through the transporter and metabolized to pharmacologically active dFdCTP that inhibits DNA synthesis and replication, leading to the antitumor efficacy

In this study, we identified favorable pharmacokinetic properties of FF-10832, including plasma stability, tumor selectivity, and prolonged maintenance of dFdCTP levels in the tumor microenvironment. The antitumor efficacy of FF-10832 is illustrated in Fig. 5. FF-10832 appeared to be extravasated from blood to tumors, possibly by EPR, and internalized in TAMs, in which the FF-10832 was processed in lysosomes to release GEM. The released drug was imported into cancer cells through the transporter and metabolized to pharmacologically active dFdCTP that inhibits DNA synthesis and replication, leading to antitumor effects. As a result, good antitumor efficacies were observed in mouse xenograft tumor models, including Capan-1, BxPC-3, and SUIT-2 tumors.

A short half-life of GEM in circulation (Fig. 2a) was probably associated with the limited pharmacological activity of this drug. A short incubation period markedly reduced the cytotoxicity of GEM in Capan-1 cells in an in vitro experiment (Fig. S3). In mice with Capan-1 tumors, an intravenous infusion of GEM at a dose of 4.4 mg/kg for 48 h exhibited antitumor activities superior to those of an intravenous bolus injection at a dose of 240 mg/kg (Fig. S4), suggesting that a longer duration of tumor cell exposure to GEM was necessary to produce effective antitumor activity. Earlier formulations of liposome-encapsulated GEM mostly did not achieve a smaller volume of distribution of GEM or longer half-life than that of unencapsulated GEM administration (14, 15, 17). These earlier formulations therefore needed to be administered frequently, twice a week or every other day, to exert their pharmacological effects in tumor-bearing mice (14, 15, 17). The pharmacologically effective doses of these formulations were often comparable with low doses of unencapsulated GEM (4–10 mg/kg) and had much lower MTDs (15, 16), suggesting that the maximum efficacies of unencapsulated GEM were not comparable with those of formulations. Thus, the construction of an efficacious liposome-encapsulated GEM formulation was considered to be a considerable challenge, possibly because of the highly hydrophilic character of GEM molecules. In contrast, FF-10832 achieved favorable pharmacokinetics with a smaller volume of distribution and a longer half-life than unencapsulated GEM (Table I) in circulating plasma.

In addition to long-term stability in circulating plasma, the amount of encapsulated drug released from the liposome formulations inside the tumor microenvironment was critical (27, 28). Thermosensitive liposomal doxorubicin and GEM, for example, can release their payloads specifically in tumor regions, but require hyperthermia to control the release rate (16, 28, 29). Drug release without a special device that produces external energy would be preferable in a clinical setting. Controlling payload release at the tumor site could lead to appreciable antitumor effects in pancreatic cancer cells. In this study, an indirect method of quantifying the in vivo release of GEM from liposomes was established, and accelerated release of GEM from the liposomes in the tumor microenvironment relative to the release rate in plasma was achieved for more than 48 h after administration of FF-10832 (Fig. 3a). The precise mechanisms underlying such GEM release remains to be elucidated, but flow cytometry and immunohistochemistry analyses revealed preferential internalization of FF-10832 by TAMs in mice with Capan-1 tumors (Figs. 2d–g). PEGylated liposomal doxorubicin, Doxil®, has been reported to be localized to the liver and spleen, which are the sites of macrophages or macrophage-like phagocytic cells, despite the STEALTH® PEG-coating strategy (30). Therefore, it can be speculated that FF-10832 also might be taken up by macrophages in tumors and other tissues. FF-10832-DiI was mainly distributed in the liver, spleen, and tumors (data not shown). A confocal laser scanning microscopy analysis revealed that a portion of FF-10832 was internalized in TAMs and localized in lysosomes (Fig. 2h). Intracellular processing of FF-10832 in lysosomes of macrophages may thus contribute, at least partially, to the release of GEM to the extracellular spaces (Figs. 3a, b), although the contribution of such release from macrophages to the overall exposure of tumor cells to GEM has not yet been quantitated. Thus, TAMs appeared to play key roles in both liposome uptake and encapsulated drug release in tumor tissues; further, TAMs possibly act as a reservoir of GEM, although various players and more complex mechanisms are speculated to be involved in anticancer pharmacology.

If TAMs are a reservoir of GEM, the toleration of TAMs to GEM exposure could be an issue, because GEM is cytotoxic to both tumor cells and proliferating normal cells. In our preliminary study, the effect of GEM on the cell viability of bone marrow-derived macrophages was investigated, and the EC_50_ value reached 10,000 nM (Fig. S5). Because the IC_50_ values of GEM in pancreatic cancer cells ranged from 18 to 28 nM (18) and the in vitro growth inhibition IC_50_ values of GEM in all pancreatic cancer cell lines examined were < 123 nM (Table S1), TAMs were considered to be able to tolerate exposure to GEM. We performed a Good Laboratory Practice (GLP) toxicity study in rats administered FF-10832 intravenously at doses ≤3 mg/kg once a week for 4 weeks. The toxicity study did not show any evidence of impaired or depleted macrophages in the liver (data not shown).

GEM is known to be cytotoxic to proliferating cells such as bone marrow cells, in addition to cancer cells. Therefore, to understand the efficiency of targeting of FF-10832 to tumors, we measured the “free” concentrations of GEM in the tumor and bone marrow, as distinct from that encapsulated in liposomes. However, it is technically difficult to measure liposome-encapsulated and unencapsulated GEM, separately in tissues. To overcome this obstacle, concentrations of dFdCTP, an active form of GEM, in tumors and bone marrow were measured. The tumor/bone marrow AUC ratio of dFdCTP after FF-10832 administration was almost eight times greater than that after GEM administration (Fig. 3c), suggesting that FF-10832 treatment has a better therapeutic index than standard or infused GEM. In the rat toxicokinetic study, the exposure to FF-10832 at the low dose (1 mg/kg) were comparable to those at the pharmacologically effective dose in mice (data not shown). In the rat GLP toxicity study, moderate to severe bone marrow hypocellularity, suggesting myelosuppression, was observed in addition to marked hematologic changes in the high-dose FF-10832 group (3 mg/kg) and the GEM group (120/80 mg/kg). The effects on the bone marrow were much milder, and were limited to bone marrow hypercellularity in the low- and mid-dose FF-10832 groups (1 and 2 mg/kg), indicating an adaptive change to mildly affected blood cells (data not shown). In the present pharmacological studies, body weight losses were observed in the FF-10832 groups, although the clinical relevance of the body weight changes was uncertain, since these experiments were conducted in immunodeficient mice bearing human tumors. Owing to the targeted delivery, fewer adverse events of myelosuppression are expected in the clinical setting.

ENT1 transports GEM, and has been reported to play a significant role in chemoresistance to pancreatic cancer (31). We therefore sought to understand the differences in sensitivity between GEM and FF-10832 in BxPC-3 and Capan-1 tumors in terms of the GEM uptake transporter ENT1, as a potential molecular mechanism for anticancer activity. ENT1 expression in BxPC-3 tumors was lower than that in Capan-1 tumors (Fig. S6), resulting in lower tumor exposures to GEM and dFdCTP in mice with BxPC3 tumors than in those with Capan-1 tumors (Table II). Longer GEM and dFdCTP exposure times could be required to achieve sufficient antitumor effects, but the survival time after the administration of unencapsulated GEM was short in mice with BxPC3 tumors (Fig. 3e). The MRT_0-last_ values of dFdCTP in mice with BxPC3 tumors after administration of FF-10832 were longer, and were comparable to those in Capan-1 tumors (Table II). By detecting EdU-positive cells, prolonged inhibition of DNA synthesis in tumors was shown in mice with BxPC3 tumors after FF-10832 administration (Fig. 4d). Thus, the sustained dFdCTP concentration profile in tumors resulted in efficacy in mice with BxPC-3 tumors and possibly overcame GEM resistance in the model (Figs. 3e, 4b). The dFdCTP concentration profiles in tumors support the predicted outcomes of GEM chemotherapy for pancreatic cancer.

We also evaluated the antitumor efficacy of FF-10832 in an orthotopic mouse model using the SUIT-2 cell line, which was derived from a human pancreatic cancer patient. The cell line carries both *KRAS* and *TP53* gene mutations, which are ubiquitous driver-gene mutations in pancreatic cancer, and are responsible for the expression of tumor biomarkers, carcinoembryonic antigens, and carbohydrate antigens 19–9 (32, 33). In a previously developed SUIT-2 orthotopic model of pancreatic adenocarcinoma in mice, we observed metastasis one week after transplantation, similar to Stage IV human pancreatic cancer (23). Drug administration starting from day 7 produced a longer median survival time in the FF-10832 group than in the GEM group (Fig. 4e), an observation which provided further evidence supporting its potential efficacy in clinical studies.

The extent of the EPR effect and its variability in preclinical cancer models, as well as in human tumors, is a key component of antitumor efficacy and the size of the therapeutic window (34, 35). Therefore, since the present study suggested the importance of the EPR effect in liposomes and uptake by TAMs, potential inter- or intra-individual heterogeneity in EPR-mediated tumor targeting may explain the heterogeneous outcomes of clinical trials. The assessment of liposome deposition in tumors, possibly by imaging technology, could identify patients well suited for FF-10832 treatment in the clinical setting. High levels of deposition of ^64^Cu-MM-302 and ferumoxytol iron nanoparticles in tumors have been reported to be associated with better treatment outcomes from HER2-targeted PEGylated liposomal doxorubicin and liposomal irinotecan, respectively, in cancer patients (35, 36).

Accelerated blood clearance (ABC), a phenomenon that has been reported to occur in PEGylated liposomal drug products, is a potential problem. The phenomenon is thought to be triggered by production of anti-PEG immunoglobulin M (IgM) upon exposure to lipids (37, 38). However, concentrations of total GEM after repeated administration of FF-10832 at 4 mg/kg were not markedly different from those after a single administration in mice (Fig. S1), indicating that there was no evidence of such a phenomenon with FF-10832. Nevertheless, the potential to induce the ABC phenomenon should be carefully monitored in clinical settings, due to potentially different susceptibilities among species.

## Conclusions

In this study, we identified favorable properties of FF-10832 as a novel liposome-encapsulated GEM. These properties included (i) high plasma stability; (ii) high tumor accumulation via the EPR effect, and at least partial internalization by TAMs; and (iii) prolonged exposure of dFdCTP in the tumor microenvironment (Fig. 5). Increased exposure to the liposomal formulation at considerably low doses of GEM achieved efficacies in mouse xenograft tumor models, including Capan-1, BxPC-3, and SUIT-2 tumors, and toleration superior to that of unencapsulated GEM, suggesting that FF-10832 is a promising candidate for the treatment of pancreatic cancer.

## Supplementary Information

ESM 1 (DOCX 56.4 kb)

ESM 2 (PNG 68 kb)

High Resolution Image (TIF 339 kb)

ESM 3 (PNG 235 kb)

High Resolution Image (TIF 995 kb)

ESM 4 (PNG 69 kb)

High Resolution Image (TIF 387 kb)

ESM 5 (PNG 109 kb)

High Resolution Image (TIF 488 kb)

ESM 6 (PNG 17 kb)

High Resolution Image (TIF 114 kb)

ESM 7 (PNG 341 kb)

High Resolution Image (TIF 1526 kb)

## Data Availability

All data generated or analyzed during this study are included in this published article and its supplementary information files.

## Abbreviations

AUC: Area under the concentration-time curve
API: Active pharmaceutical ingredient
DiI: 1,1′-dioctadecyl-3,3,3′,3′-tetramethylindocarbocyanine perchlorate
DiR: 1,1′- dioctadecyl-3,3,3′,3′-tetramethylindotricarbocyanine iodide
dFdCTP: Gemcitabine triphosphate
DLS: Dynamic light scattering
EFS: Event-free survival
ENT1: Equilibrative nucleoside transporter 1
EPR: Enhanced permeability and retention
GEM: Gemcitabine
HSPC: Hydrogenated soy phosphatidylcholine
N-MPEG-DSPE: N-(methylpolyoxyethylene oxycarbonyl)-1,2-distearoyl-sn-glycero-3-phosphoethanolamine
MRT: Mean residence time
TAM: Tumor-associated macrophages
TEM: Transmission electron microscopy;
THU: Tetrahydrouridine

## References

[1] Siegel RL, Miller KD, Jemal A (2020). Cancer statistics, 2020. CA Cancer J Clin.

[2] Vincent A, Herman J, Schulick R, Hruban RH, Goggins M (2011). Pancreatic cancer. Lancet..

[3] Burris HA 3rd, Moore MJ, Andersen J, Green MR, Rothenberg ML, Modiano MR, et al. Improvements in survival and clinical benefit with gemcitabine as first-line therapy for patients with advanced pancreas cancer: a randomized trial. J Clin Oncol. 1997;15:2403–13. 10.1200/JCO.1997.15.6.2403 9196156

[4] Federico C, Morittu VM, Britti D, Trapasso E, Cosco D (2012). Gemcitabine-loaded liposomes: rationale, potentialities and future perspectives. Int J Nanomedicine.

[5] Xu H, Paxton JW, Wu Z (2016). Development of long-circulating pH-sensitive liposomes to circumvent gemcitabine resistance in pancreatic cancer cells. Pharm Res.

[6] Mini E, Nobili S, Caciagli B, Landini I, Mazzei T (2006). Cellular pharmacology of gemcitabine. Ann Oncol.

[7] Huang P, Chubb S, Hertel LW, Grindey GB, Plunkett W (1991). Action of 2′,2′-difluorodeoxycytidine on DNA synthesis. Cancer Res.

[8] Abbruzzese JL, Grunewald R, Weeks EA, Gravel D, Adams T, Nowak B, et al. A phase I clinical, plasma, and cellular pharmacology study of gemcitabine. J Clin Oncol. 1991;9:491–8. 10.1200/JCO.1991.9.3.491 1999720

[9] Ruiz van Haperen VW, Veerman G, Boven E, Noordhuis P, Vermorken JB, Peters GJ (1994). Schedule dependence of sensitivity to 2′,2′-difluorodeoxycytidine (gemcitabine) in relation to accumulation and retention of its triphosphate in solid tumour cell lines and solid tumours. Biochem Pharmacol.

[10] Rizzuto I, Ghazaly E, Peters GJ (2017). Pharmacological factors affecting accumulation of gemcitabine′s active metabolite, gemcitabine triphosphate. Pharmacogenomics..

[11] Tempero M, Plunkett W, Ruiz Van Haperen V, Hainsworth J, Hochster H, Lenzi R (2003). Randomized phase II comparison of dose-intense gemcitabine: thirty-minute infusion and fixed dose rate infusion in patients with pancreatic adenocarcinoma. J Clin Oncol.

[12] Xie J, Yuan J, Lu L (2014). Gemcitabine fixed-dose rate infusion for the treatment of pancreatic carcinoma: a meta-analysis of randomized controlled trials. Diagn Pathol.

[13] Matsumura Y, Maeda H (1986). A new concept for macromolecular therapeutics in cancer chemotherapy: mechanism of tumoritropic accumulation of proteins and the antitumor agent smancs. Cancer Res.

[14] Paolino D, Cosco D, Racanicchi L, Trapasso E, Celia C, Iannone M, et al. Gemcitabine-loaded PEGylated unilamellar liposomes vs GEMZAR: biodistribution, pharmacokinetic features and in vivo antitumor activity. J Control Release. 2010;144:144–50. 10.1016/j.jconrel.2010.02.021 20184929

[15] Cosco D, Bulotta A, Ventura M, Celia C, Calimeri T, Perri G, et al. In vivo activity of gemcitabine-loaded PEGylated small unilamellar liposomes against pancreatic cancer. Cancer Chemother Pharmacol. 2009;64:1009–20. 10.1007/s00280-009-0957-1 19263052

[16] Tucci ST, Kheirolomoom A, Ingham ES, Mahakian LM, Tam SM, Foiret J, et al. Tumor-specific delivery of gemcitabine with activatable liposomes. J Control Release. 2019;309:277–88. 10.1016/j.jconrel.2019.07.014 PMC6815719 31301340

[17] Bornmann C, Graeser R, Esser N, Ziroli V, Jantscheff P, Keck T, et al. A new liposomal formulation of gemcitabine is active in an orthotopic mouse model of pancreatic cancer accessible to bioluminescence imaging. Cancer Chemother Pharmacol. 2008;61:395–405. 10.1007/s00280-007-0482-z 17554540

[18] Mima S, Kakinuma C, Higuchi T, Saeki K, Yamada T, Uematsu R, et al. FF-10502, an antimetabolite with novel activity on dormant cells, is superior to gemcitabine for targeting pancreatic cancer cells. J Pharmacol Exp Ther. 2018;366:125–35. 10.1124/jpet.118.248740 29653962

[19] Merriman RL, Hertel LW, Schultz RM, Houghton PJ, Houghton JA, Rutherford PG, et al. Comparison of the antitumor activity of gemcitabine and ara-C in a panel of human breast, colon, lung and pancreatic xenograft models. Investig New Drugs. 1996;14:243–7. 10.1007/BF00194526 8958178

[20] Higuchi T, Yokobori T, Takahashi R, Naito T, Kitahara H, Matsumoto T, et al. FF-10832 enables long survival via effective gemcitabine accumulation in a lethal murine peritoneal dissemination model. Cancer Sci. 2019;110:2933–40. 10.1111/cas.14123 PMC6726679 31278877

[21] Ngoune R, Peters A, von Elverfeldt D, Winkler K, Putz G (2016). Accumulating nanoparticles by EPR: a route of no return. J Control Release.

[22] ICH Q1A(R2) Guideline: stability testing of new drug substances and products (2003) International conference on harmonisation (ICH), IFPMA, Geneva, Switzerland.

[23] Higuchi T, Yokobori T, Naito T, Kakinuma C, Hagiwara S, Nishiyama M (2018). Investigation into metastatic processes and the therapeutic effects of gemcitabine on human pancreatic cancer using an orthotopic SUIT-2 pancreatic cancer mouse model. Oncol Lett.

[24] Charrois GJ, Allen TM (1663). Drug release rate influences the pharmacokinetics, biodistribution, therapeutic activity, and toxicity of pegylated liposomal doxorubicin formulations in murine breast cancer. Biochim Biophys Acta.

[25] Shipley LA, Brown TJ, Cornpropst JD, Hamilton M, Daniels WD, Culp HW (1992). Metabolism and disposition of gemcitabine, and oncolytic deoxycytidine analog, in mice, rats, and dogs. Drug Metab Dispos.

[26] Russell J, Pillarsetty N, Kramer RM, Romesser PB, Desai P, Haimovitz-Friedman A, et al. In vitro and in vivo comparison of gemcitabine and the gemcitabine analog 1-(2′-deoxy-2′-fluoroarabinofuranosyl) cytosine (FAC) in human orthotopic and genetically modified mouse pancreatic cancer models. Mol Imaging Biol. 2017;19:885–92. 10.1007/s11307-017-1078-6 PMC5696795 28349292

[27] Allen TM, Cullis PR (2004). Drug delivery systems: entering the mainstream. Science.

[28] Ait-Oudhia S, Mager DE, Straubinger RM (2014). Application of pharmacokinetic and pharmacodynamic analysis to the development of liposomal formulations for oncology. Pharmaceutics..

[29] Yarmolenko PS, Zhao Y, Landon C, Spasojevic I, Yuan F, Needham D, et al. Comparative effects of thermosensitive doxorubicin-containing liposomes and hyperthermia in human and murine tumours. Int J Hyperth. 2010;26:485–98. 10.3109/02656731003789284 PMC2956508 20597627

[30] Gabizon A, Shmeeda H, Barenholz Y (2003). Pharmacokinetics of pegylated liposomal doxorubicin: review of animal and human studies. Clin Pharmacokinet.

[31] Andersson R, Aho U, Nilsson BI, Peters GJ, Pastor-Anglada M, Rasch W, et al. Gemcitabine chemoresistance in pancreatic cancer: molecular mechanisms and potential solutions. Scand J Gastroenterol. 2009;44:782–6. 10.1080/00365520902745039 19214867

[32] Iwamura T, Taniguchi S, Kitamura N, Yamanari H, Kojima A, Hidaka K, et al. Correlation between CA19-9 production in vitro and histological grades of differentiation in vivo in clones isolated from a human pancreatic cancer cell line (SUIT-2). J Gastroenterol Hepatol. 1992;7:512–9. 10.1111/j.1440-1746.1992.tb01030.x 1391733

[33] Moore PS, Sipos B, Orlandini S, Sorio C, Real FX, Lemoine NR, et al. Genetic profile of 22 pancreatic carcinoma cell lines. Analysis of K-ras, p53, p16 and DPC4/Smad4. Virchows Arch. 2001;439:798–802. 10.1007/s004280100474 11787853

[34] Kalra AV, Kim J, Klinz SG, Paz N, Cain J, Drummond DC, et al. Preclinical activity of nanoliposomal irinotecan is governed by tumor deposition and intratumor prodrug conversion. Cancer Res. 2014;74:7003–13. 10.1158/0008-5472.CAN-14-0572 25273092

[35] Lee H, Shields AF, Siegel BA, Miller KD, Krop I, Ma CX (2017). (64)Cu-MM-302 Positron emission tomography quantifies variability of enhanced permeability and retention of nanoparticles in relation to treatment response in patients with metastatic breast cancer. Clin Cancer Res..

[36] Ramanathan RK, Korn RL, Raghunand N, Sachdev JC, Newbold RG, Jameson G, et al. Correlation between ferumoxytol uptake in tumor lesions by MRI and response to nanoliposomal irinotecan in patients with advanced solid tumors: a pilot study. Clin Cancer Res. 2017;23:3638–48. 10.1158/1078-0432.CCR-16-1990 28159813

[37] Dams ET, Laverman P, Oyen WJ, Storm G, Scherphof GL, van Der Meer JW (2000). Accelerated blood clearance and altered biodistribution of repeated injections of sterically stabilized liposomes. J Pharmacol Exp Ther.

[38] Mohamed M, Abu Lila AS, Shimizu T, Alaaeldin E, Hussein A, Sarhan HA, et al. PEGylated liposomes: immunological responses. Sci Technol Adv Mater. 2019;20:710–24. 10.1080/14686996.2019.1627174 PMC6598536 31275462

