# Supplementary material for: A Liposomal Gemcitabine, FF-10832, Improves Plasma Stability, Tumor Targeting, and Antitumor Efficacy of Gemcitabine in Pancreatic Cancer Xenograft Models
Source: Pharm Res. 2021 May 7;38(6):1093–106. doi: 10.1007/s11095-021-03045-5 (PMC8217058; doi:10.1007/s11095-021-03045-5)
Supplement: Supplementary file 1 — (DOCX 56.4 kb) [file 11095_2021_3045_MOESM1_ESM.docx]

**Supplementary Materials** **for**

**A Liposomal Gemcitabine, FF-10832, Improves Plasma Stability, Tumor Targeting, and Antitumor Efficacy of Gemcitabine in Pancreatic Cancer Xenograft Model**

Journal Name: Pharmaceutical Research

Takeshi Matsumoto,^1^ Takashi Komori,^1^ Yuta Yoshino,^1^ Tadaaki Ioroi,^1^ Tsukasa Kitahashi,^1^ Hiromu Kitahara,^1^ Kohei Ono,^1^ Tamami Higuchi,^1^ Masayo Sakabe,^1^ Hiroshi Kori,^1^ Masahiro Kano,^1^ Etsuko Hori,^2^ Yukio Kato,^3^ Shinji Hagiwara^1^

^1^Bioscience and Engineering laboratories, FUJIFILM Corporation, 577 Ushijima, Kaisei-machi, Ashigarakami-gun Kanagawa 258-8577, Japan

^2^Analysis Technology Center, FUJIFILM Corporation, Nakanuma 210, Minamiashigara-shi, Kanagawa-ken, 250-0193, Japan

^3^Faculty of Pharmacy, Institute of Medical, Pharmaceutical and Health Sciences, Kanazawa University

**Corresponding author:** Takeshi Matsumoto,

Bioscience and Engineering laboratories, FUJIFILM Corporation., 577 Ushijima, Kaisei-machi, Ashigarakami-gun Kanagawa 258-8577, Japan

Phone: (+81)-465-86-1707; Fax: (+81)-465-86-1224

E-mail: [takeshi.matsumoto@fujifilm.com](mailto:takeshi.matsumoto@fujifilm.com)

**Supplementary Methods**

**Preparation of FF-10832**

Cholesterol, hydrogenated soy phosphatidylcholine (HSPC), and N-(methylpolyoxyethylene oxycarbonyl)-1,2-distearoyl-*sn*-glycero-3-phosphoethanolamine sodium salt (N-MPEG-DSPE) at the molar ratio of 4:15:1 were dissolved in an organic solvent containing ethanol and ethyl acetate. Water phase (pH 7–8) was prepared by using disodium hydrogen phosphate 12-hydrate and sodium dihydrogen phosphate dihydrate. The water phase and lipid solution were mixed at a volume ratio of 8:3. Next, the mixture was dispersed to form particles with approximately 70 nm by heating above the phase-transition temperature. Thereafter, the organic solvent was removed by heating, and the external phase of the liposomes was replaced with 0.09% sodium chloride solution by diafiltration to obtain empty liposomes. Phosphate-buffered solution (PBS) was prepared by dissolving sodium chloride (81.6 g), disodium hydrogen phosphate 12-hydrate (29.0 g), and sodium dihydrogen phosphate dehydrate (2.29 g) in water (948 g). Gemcitabine (GEM) hydrochloride, PBS, water, and sodium hydroxide were mixed and heated to dissolve GEM hydrochloride. Subsequently, the drug solution and empty liposomes were mixed, and the pH was adjusted to be neutral with sodium hydroxide. The drug–lipid mixture was heated to above the phase-transition temperature to facilitate GEM loading into the internal phase of the liposomes. Next, residual GEM in the external phase was removed and replaced with 9.4% sucrose/10 mM histidine solution by diafiltration. The purified liposomes were sterile-filtered to obtain FF-10832. For preparation of FF-10832 labeled with fluorescent dyes of DiI and DiR (FF-10832-DiI and FF-10832-DiR, respectively), DiI or DiR that was 0.31 % (w/w) of HSPC was added to the lipid solution, and the subsequent manufacturing process was the same as that used for FF-10832.

**Bone Marrow-derived Macrophages and Cell Lines**

Bone marrow-derived macrophages were obtained from femur of female BALB/c mice (Charles River Laboratories, Kanagawa, Japan). Briefly, the femur was centrifuged (1,800 g, 10 sec., 4°C) and bone marrow cells were collected. The cells were treated with lysing buffer (×10 diluted BD Pharm Lyse^TM^, BD Biosciences, Franklin Lakes, NJ) and pelleted by centrifugation (300 × g, 10 min, 4°C). After removing the supernatant, the cells were resuspended in RPMI 1640 (Life Technologies, Waltham, MA) containing with 10% fetal bovine serum (FBS), 1% Penicillin-Streptomycin (Gibco, Waltham, MA), and 20 ng/mL M-CSF (PEPROTECH, Cranbury, NJ). The suspension was incubated in T225 Flask at 37°C and 5% CO_2_ for 6 days with addition of fresh medium after 3 days to obtain macrophage monolayers.

Capan-1, Capan-2, HPAF-II, and BxPC-3 were obtained from the American Type Culture Collection (Manassas, VA). SUIT-2 and MiaPaca-2 cells were obtained from the JCRB cell bank (Osaka, Japan). Panc-1 cells were obtained from RIKEN BioResource Research Center (Ibaraki, Japan). Capan-1 cells were cultured in Iscove's Modified Dulbecco's Medium (Life Technologies) containing 20% FBS (Gibco) and 1% Penicillin-Streptomycin (Gibco). The culture medium for Capan-2 cells was McCoy’s 5A (Life Technologies) containing 10% FBS and 1% PenStrep. The culture medium for HPAF-II and MiaPaca-2 cells was Minimum Essential Medium (Life Technologies) containing 10% FBS and 1% Penicillin-Streptomycin. The culture medium for BxPC-3, SUIT-2, and Panc-1 cells was RPMI 1640 medium containing 10% FBS and 1% Penicillin-Streptomycin. These cells were cultured in a 37°C incubator with 5% CO_2_.

**Qualification of GEM, Gemcitabine Triphosphate (dFdCTP), DiI, and Lipid**

Total and unencapsulated GEM was analyzed by HPLC (Acquity UPLC H-Class system, Waters, Milford, MA). Chromatography was performed using Waters Acquity UPLC HSS T3 column (2.1 mm × 5 cm with a particle size of 1.8 μm, Waters). The mobile phases used were phosphate buffer (Buffer A, pH 2.4 to 2.6) and methanol (Buffer B).

Total lipids were analyzed by HPLC-CAD (Ultimate3000RS system, Thermo Fisher Scientific Inc.). The instrument was equipped with a pump (DGP-3000RS), an autosampler (WPS-3000TRS), column compartment (TCC-3000RS), DAD (DAD-3000RS), and CAD (Corona Veo RS). Chromatography was performed using Waters Acquity UPLC BEH C18 column (2.1 mm × 15 cm with a particle size of 1.7 μm, Waters). The mobile phase used was 2.5 mM ammonium acetate dissolved in methanol. For the Corona Veo RS, the gas evaporation temperature was adjusted to 50°C, and data acquisition rate was set to 5 Hz.

To measure the GEM and dFdCTP concentrations in tissues, a 4-fold volume of methanol containing the internal standard was added to the tissues to be examined. The samples were vortex-mixed for 3 min and immediately frozen in liquid nitrogen. The frozen tissues were homogenized (3,000 rpm for 10 sec, 10 times) with a MicroSmashTM (TOMY SEIKO, Tokyo, Japan), centrifuged (13,000 rpm for 3 min), and the supernatant was analyzed by liquid chromatography/tandem mass spectrometry (LC/MS/MS) using a triple-quadrupole mass spectrometer with electrospray ionization (AB Sciex. Triple Quad 5500 Foster City, CA) coupled to an ultra-performance liquid chromatography system (ACQUITY UPLC system, Waters). The multiple reaction monitor was set at 264.08/112.1 for GEM and 503.87/326.3 for dFdCTP. Chromatography was performed by using an Inertsustain Swift C18 column (particle size, 1.9 μm; 2.1 × 50 mm, GL Sciences, Tokyo, Japan).

The contents of DiI in plasma and tumor tissues were analyzed by RP-HPLC (SHIMADZU LabSolutions Nexera series, SHIMADZU, Kyoto, Japan). Chromatography was performed by using a Waters Acquity BEH C8 column (particle size, 1.7 μm, Waters).

**Pharmacokinetic Parameters**

The pharmacokinetic parameters of GEM and dFdCTP were determined by noncompartmental analysis using WinNonLin (version 6.4; Pharsight Corporation, Mountain View, CA). The area under the concentration time curve (AUC) up to the last measured concentration time point (AUC_0–last_) was calculated by using the trapezoidal method. Total clearance (CL), initial volume of distribution (V_0_), initial volume of distribution at steady-state (Vd_ss_), mean residence time (MRT_0–last_), and overall half-life (t_1/2_) were estimated by using moment analysis of the plasma concentration profiles of GEM.

**Determination of mRNA Expression Level in Tumors**

Total RNA was purified from BxPC-3 and Capan-1 tumors by using an RNeasy Plus Mini Kit (Qiagen, Mettmann, DE). From the isolated RNA, 400 ng was used for cDNA generation using a High-Capacity RNA-to-cDNA kit (Thermo Fisher Scientific Inc.). cDNAs were mixed with TaqMan Gene Expression Master Mix (Applied Biosystems, Waltham, MA) and Taqman Probes (Applied Biosystems), and real time-polymerase chain reaction was performed by using a qPCR MX 3000 (Agilent Technologies, Santa Clara, CA). The amount of amplified mRNA was analyzed for each target gene by using MxPro Mx3000P version 4.10 software. The CT value was detected by using the amplification-based threshold method from the mRNA amplification curve. mRNA expression in each group was compared by using the comparative Ct method (ΔΔCt method).

**Detection of DNA Synthesis in Tumors**

FF-10832 (4 mg/kg), GEM (240 mg/kg), or vehicle (PBS) was intravenously administered to mice with Capan-1 or BxPC3 tumors (n = 3 animals/group) via the tail vein. Tumors were collected 4, 24, 48, and 72 h after treatment. Prior to tumor collections, 50 mg/kg of EdU (Thermo Fisher Scientific Inc.) was intraperitoneally administered 3 h before. The collected tumors were embedded in OCT compound (Sakura Finetek Japan, Tokyo, Japan) and immediately frozen at −80°C. The frozen samples were sectioned into 5-µm slices by using a Cryotome FSE (Thermo Fisher Scientific Inc.), which was mounted on slides. The sections were fixed with 10% formalin neutral buffer solution (FUJIFILM Wako Pure Chemical, Tokyo, Japan) and permeabilized with 0.5% Triton X-100 (MP Biomedicals, Santa Ana, CA), followed by EdU staining and the nuclei by using a Click-iT EdU Alexa Fluor 488 Imaging Kit (Thermo Fisher Scientific Inc.) and Hoechst 33342, respectively. Tumor images were captured by using a confocal quantitative image cytometer CQ1 (Yokogawa Electric Corporation, Tokyo, Japan) and analyzed by CQ1 measurement software. The captured areas consisted of three randomly selected fields (× 100) per tumor specimen. The percentages of DNA-synthesizing cells were calculated according to the following formula:

DNA-synthesizing cells (%) = EdU-positive nuclei counts / Hoechst 33342-positive nuclei counts × 100

**Supplementary Figures**

**Fig. S1. Plasma Concentration Profiles of GEM after Repeated Administration of FF-10832**

Female BALB/cAJcl-nu/nu mice were intravenously administered 4 mg/kg of FF-10832 once a week for 3 weeks, and plasma concentrations of GEM are shown. Blood (600 µL) was collected in tubes containing THU (100 µg/mL in blood) at 0.25, 2, 4, 8, 24, and 48 h after the first administration of FF-10832 and at 0.25, 24, and 48 h after the second/third administration of FF-10832, and centrifuged (800 × g, 4°C, 10 minutes) to prepare plasma samples for measuring total GEM. After blood sampling, animals were euthanized by exsanguinations under anesthesia. Portions of the plasma samples (approximately 300 µL) were loaded onto an ultrafilter (Amicon^®^ Ultra-0.5, MWCO:10K) and centrifuged (3,400 × g, 4°C, 5 or 10 min) to prepare plasma ultrafiltrate samples for measuring free GEM. Concentrations of GEM were analyzed by using LC/MS-MS as described in the Supplementary Methods. Each data point represents the mean ± standard deviation (n = 4 animals/group).

**Fig. S2. Confocal Laser Scanning Microscopy in Peritoneal Macrophages**

Male Jcl:ICR (CLEA Japan) were intraperitoneally administered 5 mg/kg of FF-10832-DiI. Three hours after administration, 10 mL of ice-cold PBS was administered into the peritoneal cavity, and peritoneal fluids were collected for isolation. After washing twice with PBS followed by centrifugation at 400 × g for 5 min at 4°C, the cells were suspended in RPMI 1640 medium containing 10% FBS and 1% Penicillin-Streptomycin, seeded (2 × 10^5^ cells/well) into a 96-well plate (CellCarrier-96), and stained for 30 min with 50 nM LysoTracker Deep Red. Then, the cells were stained for 10 min with 10 μg/mL Hoechst 33342 followed by further incubation in PBS. The cell images were captured by using a confocal quantitative image cytometer CQ1 and analyzed by CQ1 measurement software. Uptake of FF-10832-DiI is shown by CLSM images for nuclei (blue), DiI (red), lysosome (green), and merged.

**Fig. S3. In Vitro Effects on Capan-1 Cell viability**

Capan-1 cells were in vitro exposed to GEM at concentrations of 1–10,000 nM for 18 min, 3 h, 24 h, and 72 h, and GEM was removed with washes followed by further incubation until 120h after the start of the GEM treatment. Cell viability was evaluated by performing the CellTiter-Glo^Ⓡ^ cell assay (Promega Corporation, Madison, WI). Each data point represents mean ± standard deviation (n = 3).

**Fig. S4. Antitumor Efficacies after Intravenous Bolus Injections and Subcutaneous Continuous Infusions of Unencapsulated GEM in Mice with Capan-1 Tumors**

After randomization, mice with Capan-1 tumors (8 animals / group) received intravenous injections of GEM at 240 mg/kg or subcutaneous 48-h continuous infusions (1 μL/h) of GEM at 2.2, 4.4, or 6.6 mg/kg with a mini-osmotic pump (1003D, DURECT Corp, Cupertino, CA) that had been subcutaneously implanted under sterile conditions with loading 100 μL of GEM solutions, which were 1, 2, and 3 mg/mL for the 2.2, 4.4, and 6.6 mg/kg groups, respectively, once a week for 3 weeks. Tumor volumes were measured over time, which are presented as the mean ± standard deviation. Body-weight losses >20% were observed in three and eight of eight animals in the 4.4 and 6.6 mg/kg/48 h groups, respectively, exceeding the MTD, so the administration in these animals was discontinued.

**Fig. S5. In vitro Effects on Bone Marrow-Derived Macrophage Viability**

Bone marrow-derived macrophages were exposed to GEM at concentrations from 4.6–10,000 nM for 72 h, and then cell viability was evaluated by performing the ATP assay. Each data point represents the mean ± standard deviation (n = 6).

**Fig. S6. mRNA Expressions of Nucleoside Transporters and Enzymes in Capan-1 and BxPC-3 Tumors**

Capan-1 tumors (blue) and BxPC-3 tumors (red) were collected when the average tumor volume reached approximately 100–500 mm in mice with Capan-1 and BxPC-3 tumors. mRNA expressions in each tumor of ENT1 (a), ENT2 (b), deoxycytidine kinase (c), and cytidine deaminase (d) were determined. The relative changes from the mean values in Capan-1 tumors are presented as the mean ± standard deviation (n = 3).

**Supplementary Table**

**Table S1. In vitro Pancreatic Cancer Cell Viability after Treatment with GEM for Various Periods** ^a)^

| Time (h) | 0.3 | 1 | 3 | 8 | 24 | 33 | 48 | 120 |
| --- | --- | --- | --- | --- | --- | --- | --- | --- |
| Capan-1 | N.D. | 1,802 | 299 | 103 | 21 | 14 | 10 | 11 |
| Capan-2 | N.D. | N.D. | N.D. | N.D. | 1,560 | 466 | 123 | 71 |
| HPAF-2 | N.D. | N.D. | 501 | 124 | 29 | 21 | 18 | 15 |
| SUIT-2 | 2,110 | 290 | 79 | 30 | 10 | 6 | 5 | 5 |
| BxPC-3 | N.D. | N.D. | 891 | 204 | 27 | 19 | 8 | 10 |
| MiaPaca-2 | N.D. | N.D. | N.D. | 453 | 91 | 45 | 28 | 28 |
| Panc-1 | N.D. | N.D. | 864 | 138 | 68 | 38 | 18 | 21 |

^a)^ Pancreatic cancer cells, including Capan-1, Capan-2, HPAF-2, SUIT-2, BxPC-3, MiaPaca-2, and Panc-1 cells, were exposed in vitro to GEM at concentrations of 1–10,000 nM for 18 min, 1, 3, 8, 24, 33, 48 h, and 120 h, and GEM was removed with washes followed by further incubation until 120 h after start of the GEM treatment. Cell viability was evaluated by using the CellTiter-Glo^®^ assay (Promega Corporation). Each point represents the mean values (n = 3).
