# Supplementary figures and images for: A Liposomal Gemcitabine, FF-10832, Improves Plasma Stability, Tumor Targeting, and Antitumor Efficacy of Gemcitabine in Pancreatic Cancer Xenograft Models
Source: Pharm Res. 2021 May 7;38(6):1093–106. doi: 10.1007/s11095-021-03045-5 (PMC8217058; doi:10.1007/s11095-021-03045-5)

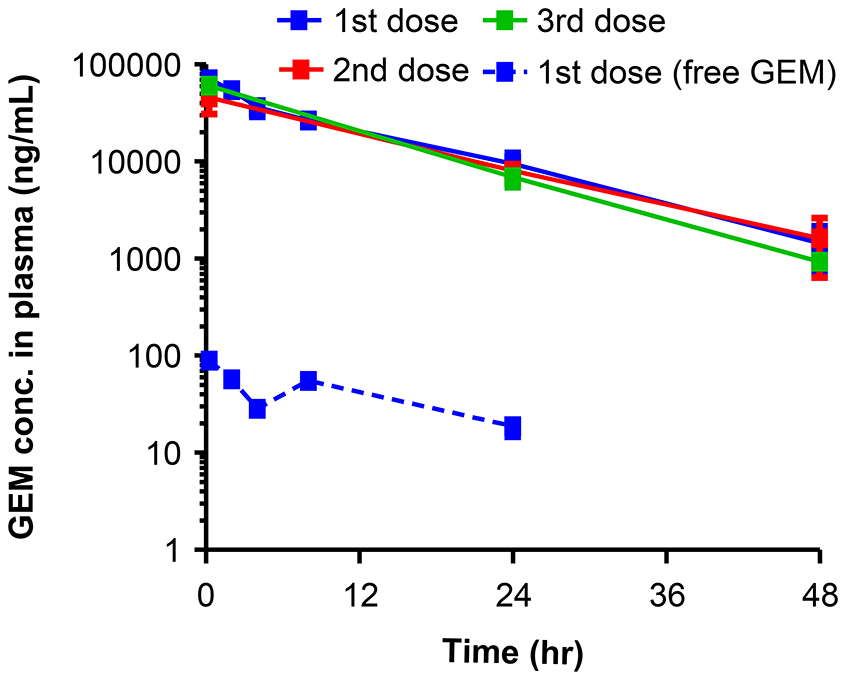

Supplement: Supplementary file 2 — (PNG 68 kb) [file 11095_2021_3045_Fig6_ESM.png]

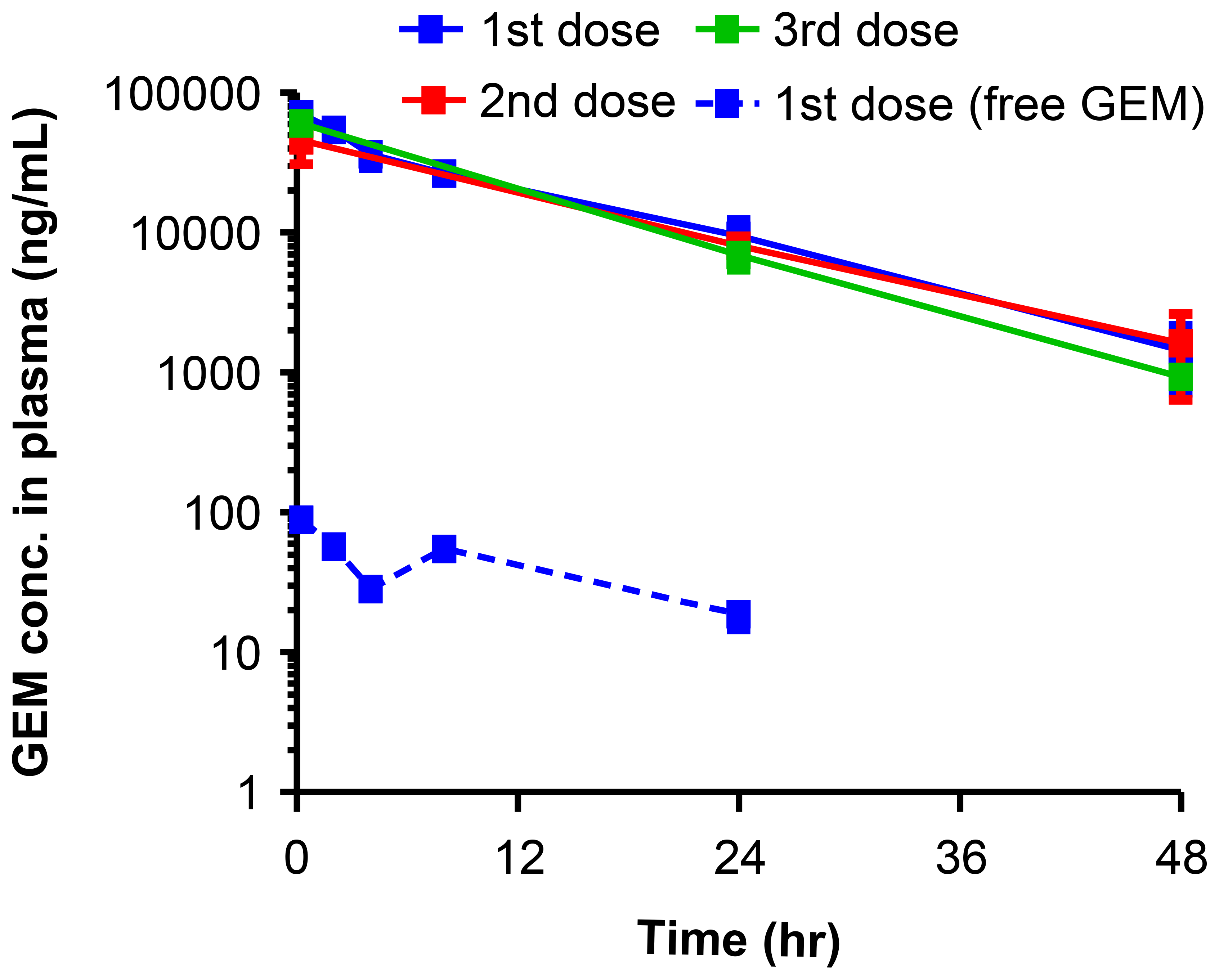

Supplement: Supplementary file 3 — High Resolution Image (TIF 339 kb) [file 11095_2021_3045_MOESM2_ESM.tif]

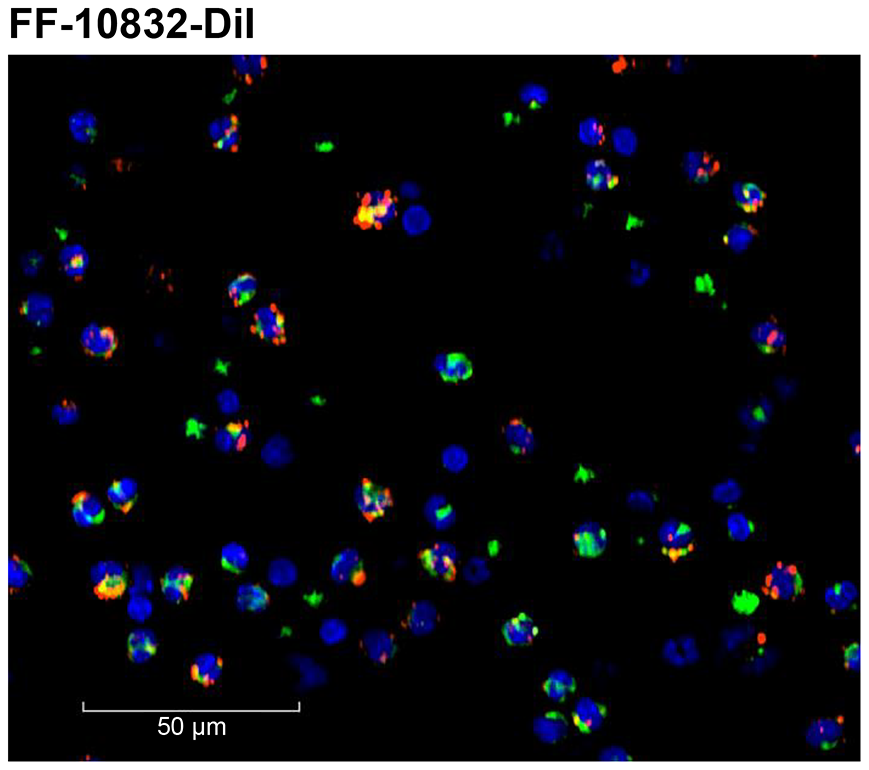

Supplement: Supplementary file 4 — (PNG 235 kb) [file 11095_2021_3045_Fig7_ESM.png]

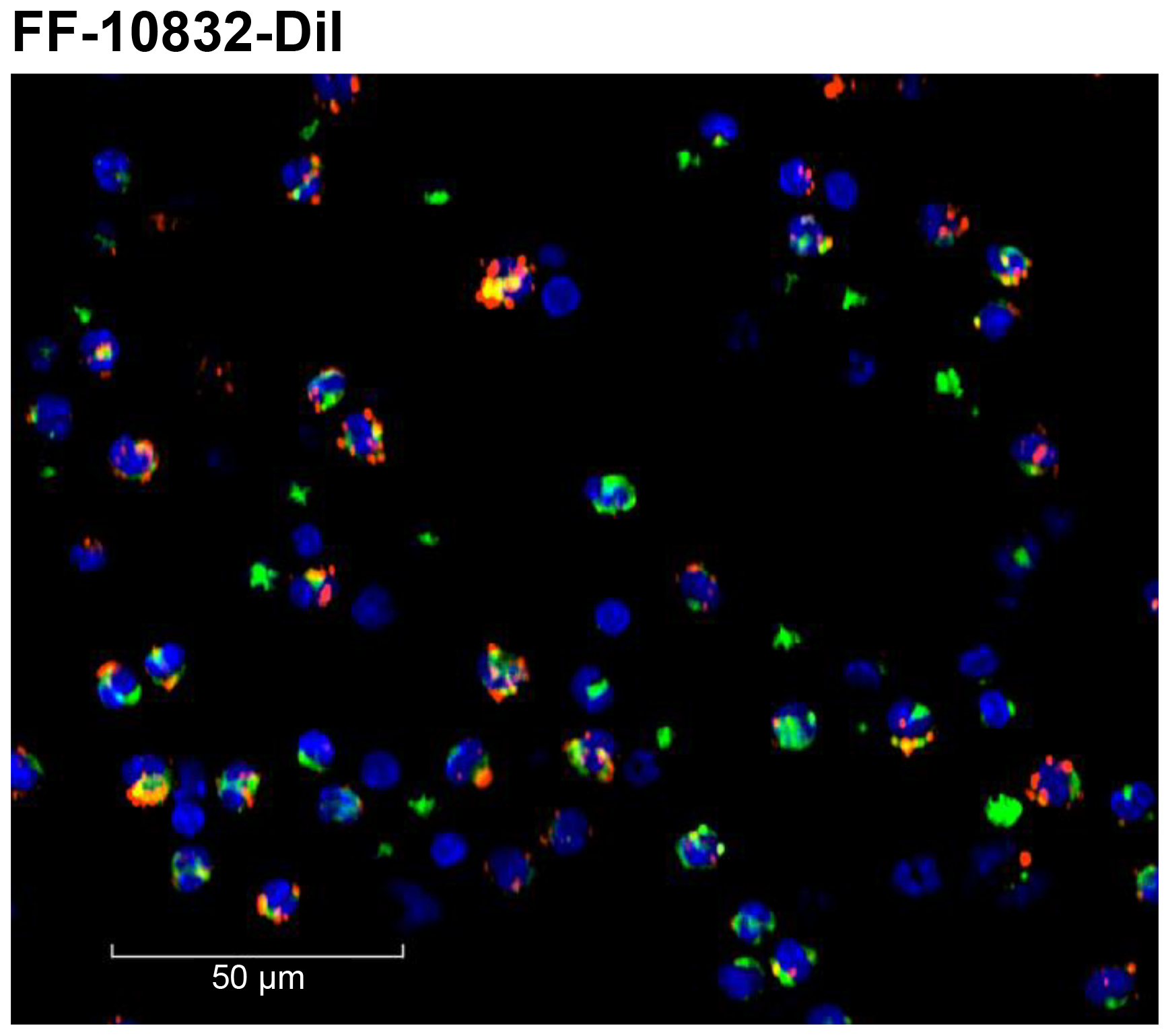

Supplement: Supplementary file 5 — High Resolution Image (TIF 995 kb) [file 11095_2021_3045_MOESM3_ESM.tif]

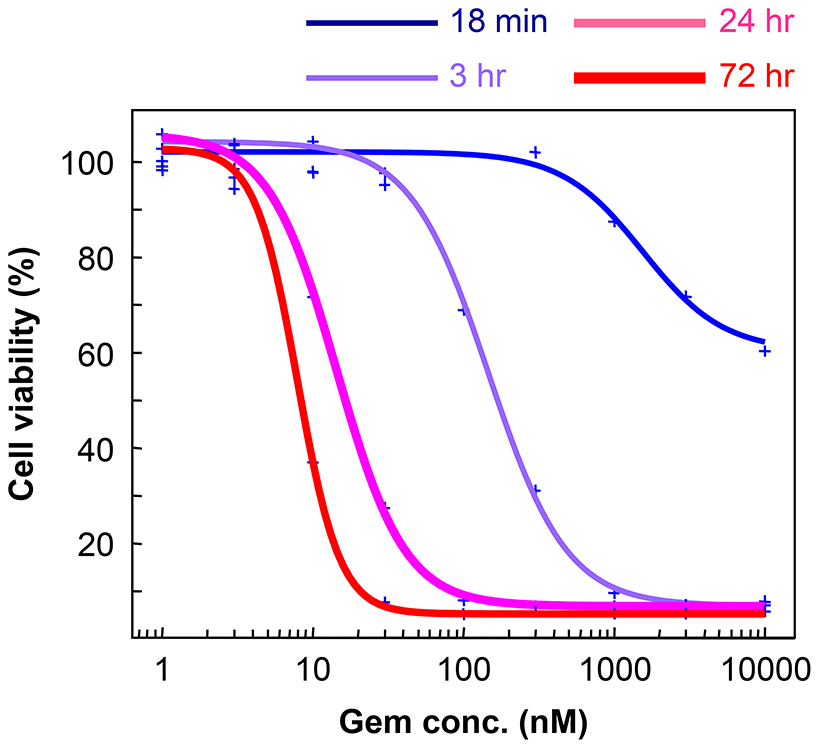

Supplement: Supplementary file 6 — (PNG 69 kb) [file 11095_2021_3045_Fig8_ESM.png]

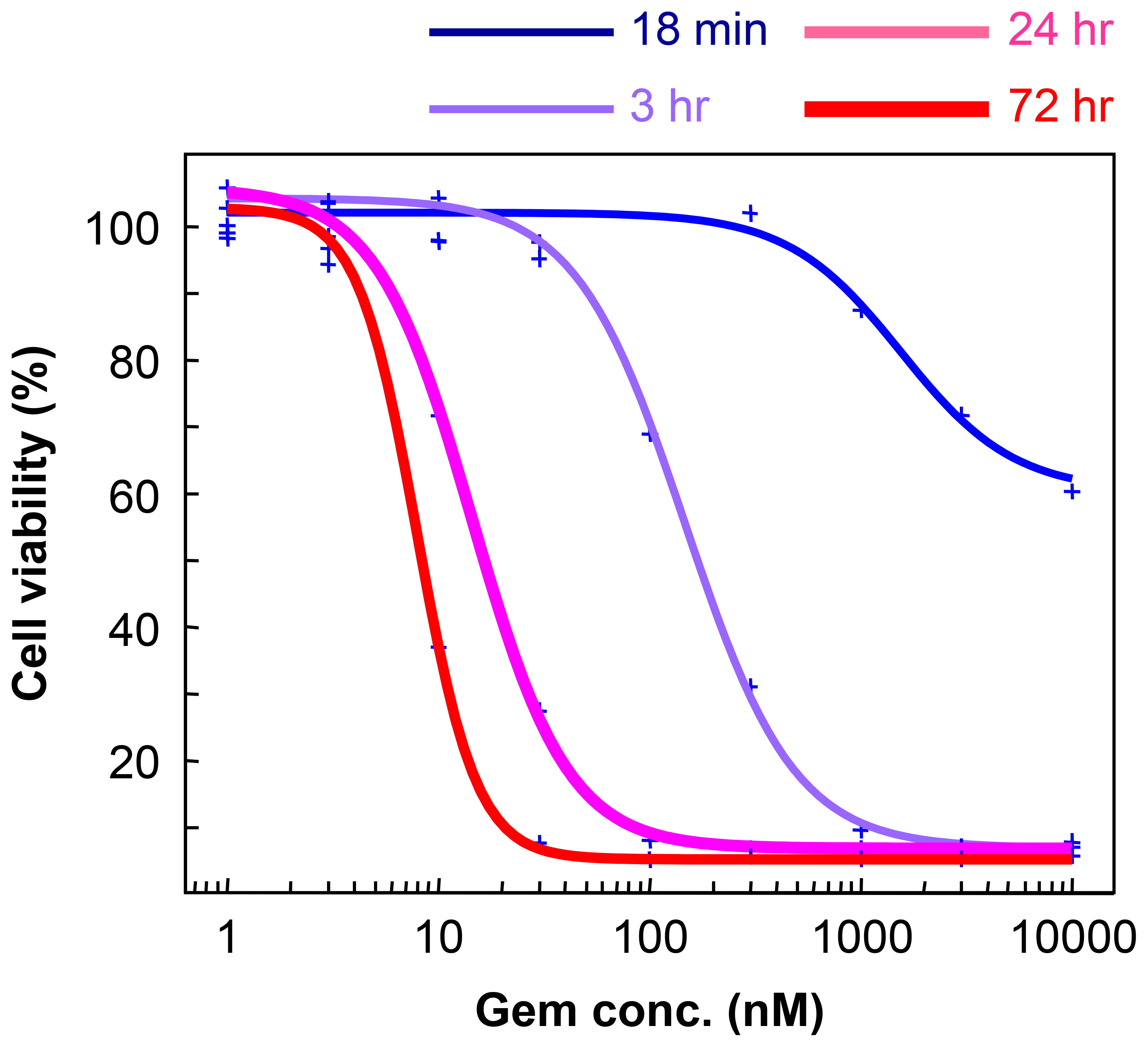

Supplement: Supplementary file 7 — High Resolution Image (TIF 387 kb) [file 11095_2021_3045_MOESM4_ESM.tif]

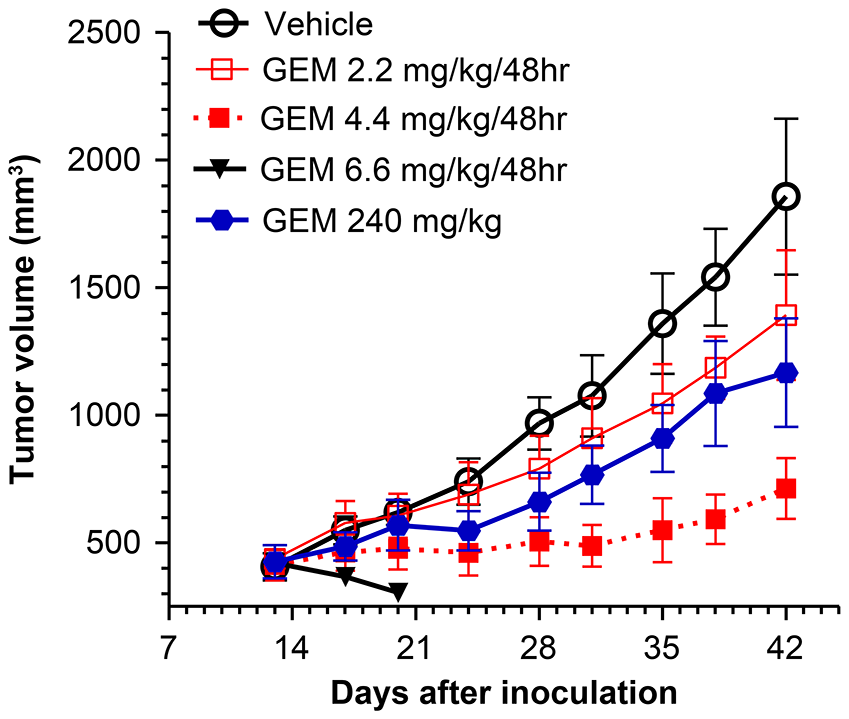

Supplement: Supplementary file 8 — (PNG 109 kb) [file 11095_2021_3045_Fig9_ESM.png]

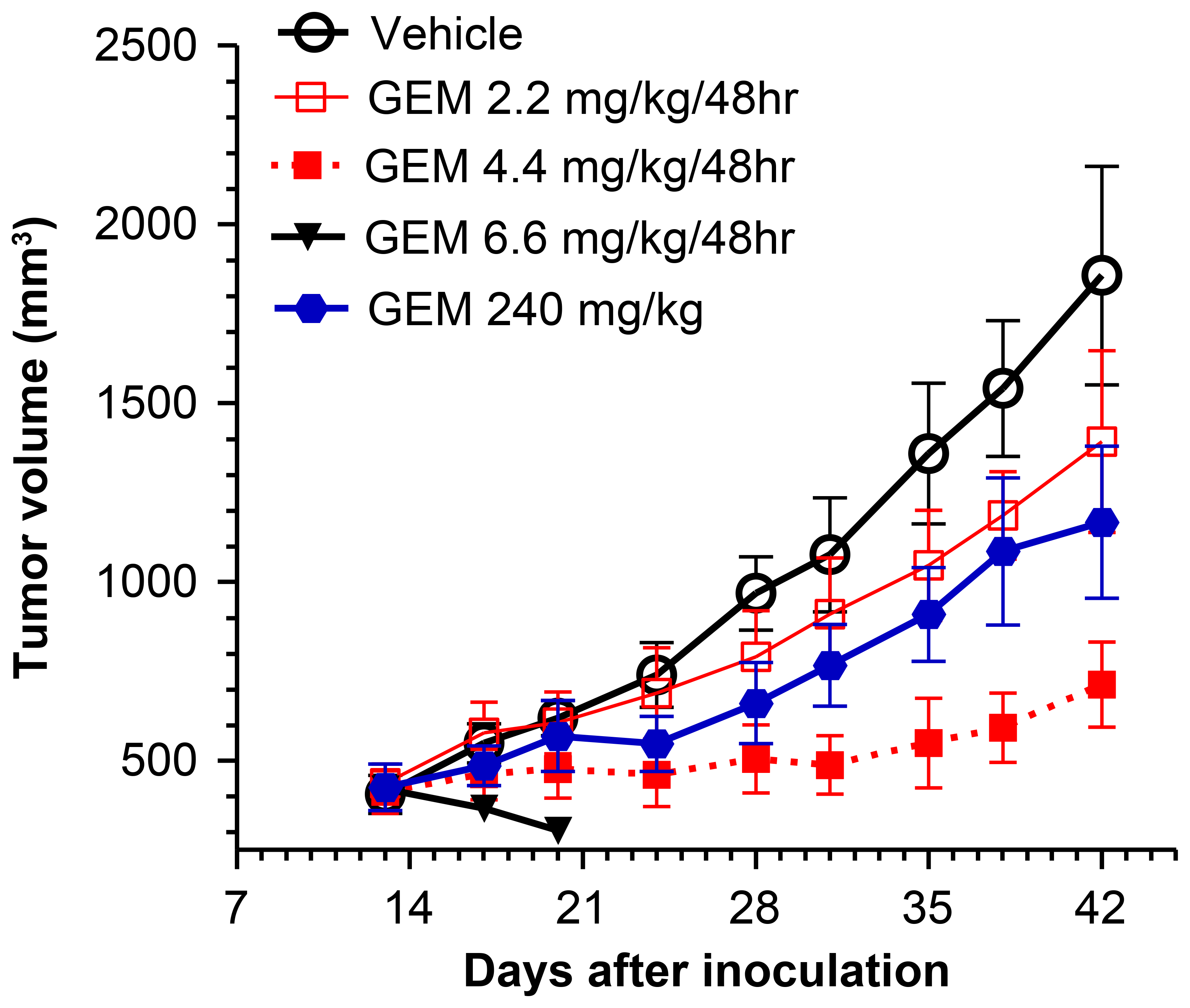

Supplement: Supplementary file 9 — High Resolution Image (TIF 488 kb) [file 11095_2021_3045_MOESM5_ESM.tif]

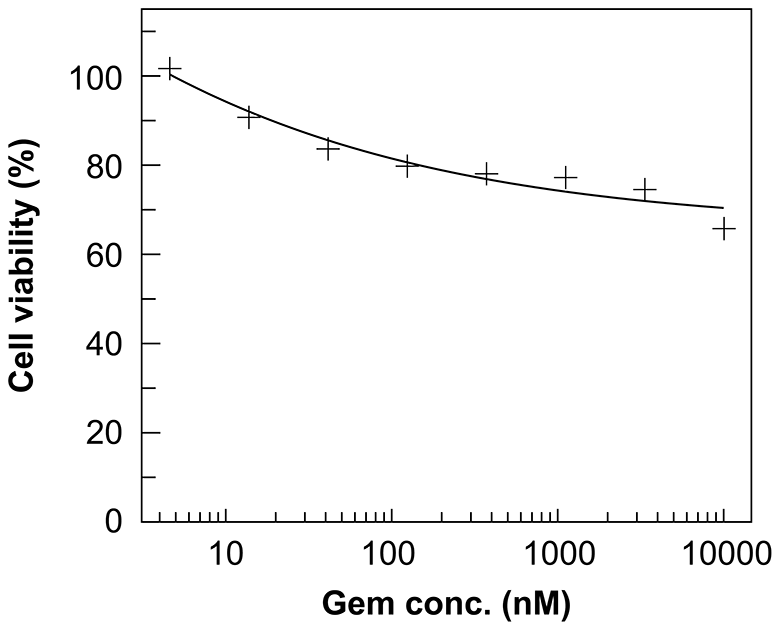

Supplement: Supplementary file 10 — (PNG 17 kb) [file 11095_2021_3045_Fig10_ESM.png]

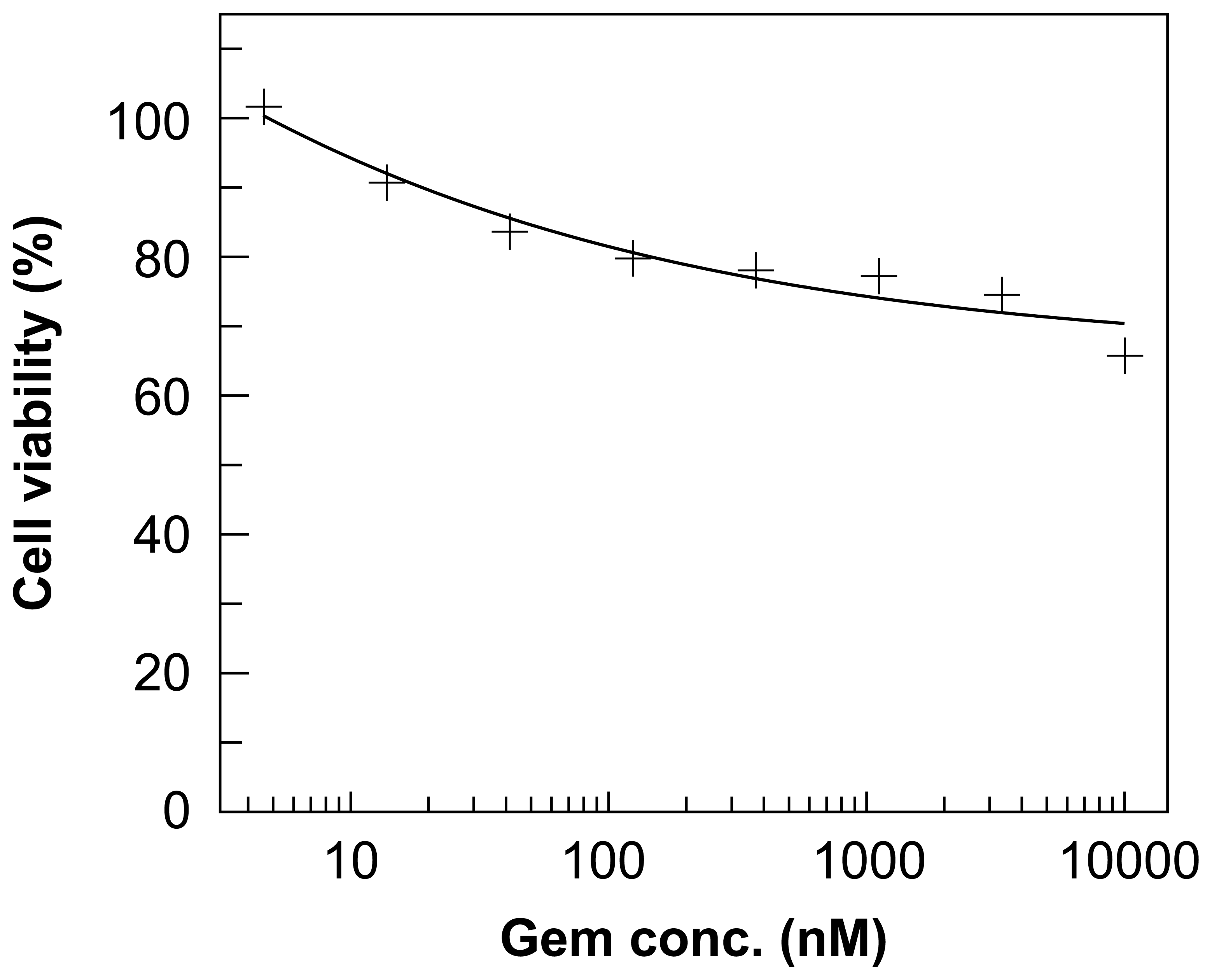

Supplement: Supplementary file 11 — High Resolution Image (TIF 114 kb) [file 11095_2021_3045_MOESM6_ESM.tif]

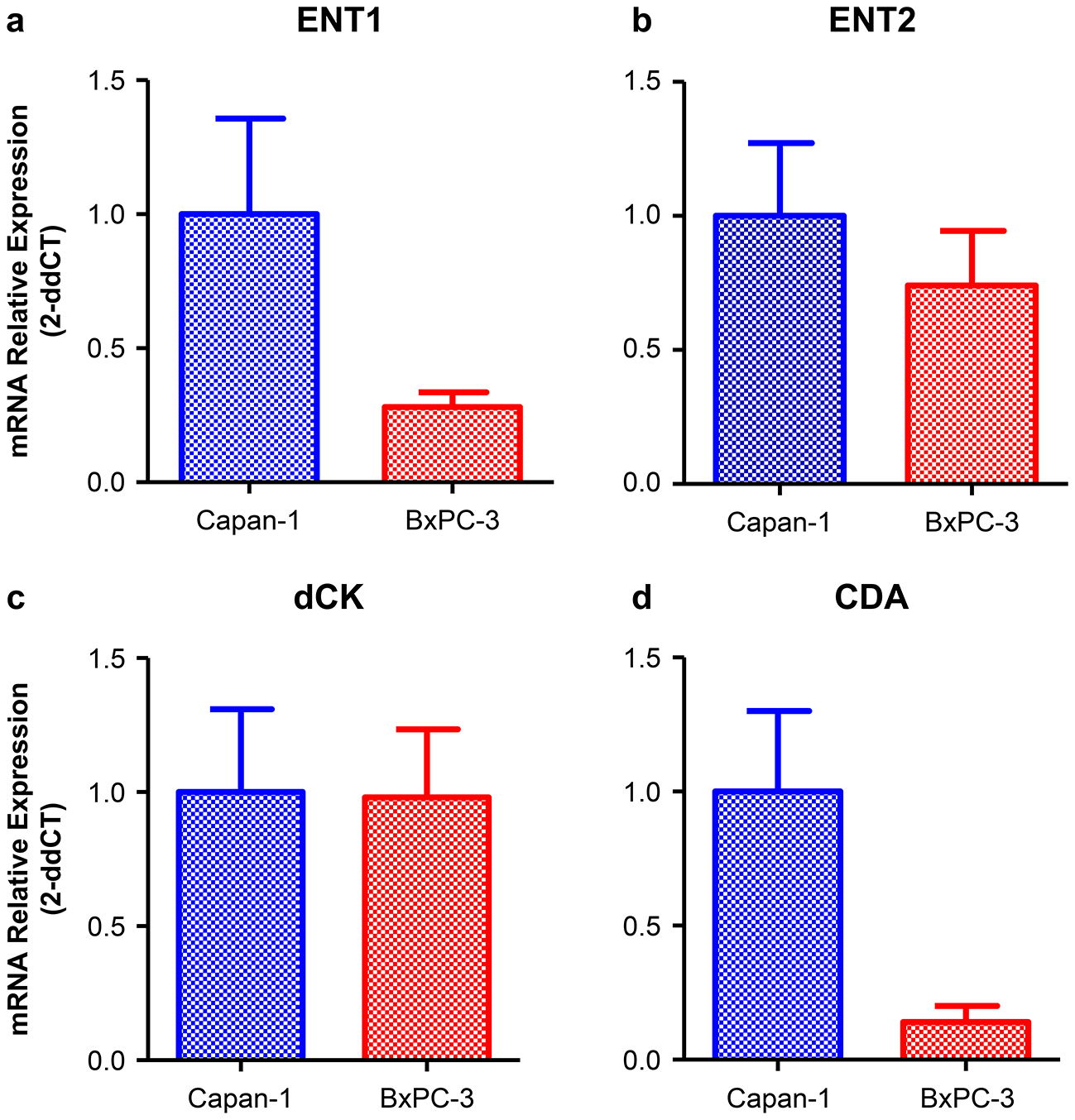

Supplement: Supplementary file 12 — (PNG 341 kb) [file 11095_2021_3045_Fig11_ESM.png]

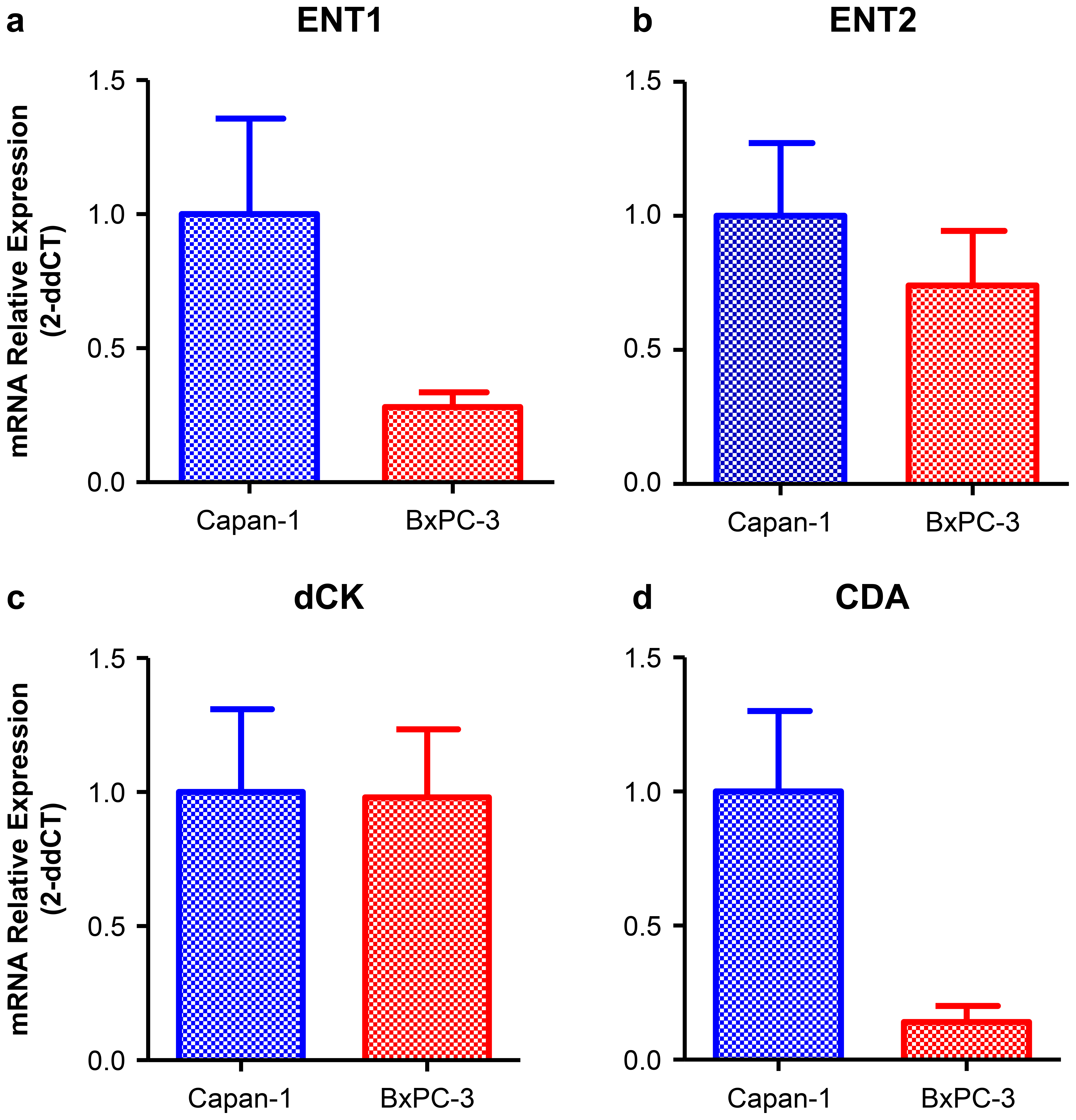

Supplement: Supplementary file 13 — High Resolution Image (TIF 1526 kb) [file 11095_2021_3045_MOESM7_ESM.tif]
